# Quality of Cancer-Related Clinical Coding in Primary Care in North Central London: Mixed Methods Quality Improvement Project

**DOI:** 10.2196/73205

**Published:** 2026-01-07

**Authors:** Afsana Bhuiya, Graham Roberts, Katie Tucker, Stefanie Bonfield, Georgia Black

**Affiliations:** 1 North Central London Cancer Alliance London United Kingdom; 2 Wolfson Institute of Population Health Queen Mary University of London London United Kingdom

**Keywords:** cancer diagnosis, cancer pathway, cancer risk factors, cancer treatment, cancer, clinical coding, coding completeness, coding data, coding diversity, coding processes, coding quality, coding validation, coding variation, inequalities data, primary care coding, quality improvement, SNOMED CT, Systematized Nomenclature of Medicine – Clinical Terms

## Abstract

**Background:**

The North Central London (NCL) Cancer Alliance carried out a quality improvement (QI) project to fill a distinct knowledge gap regarding the quality of clinical coded data in a primary care electronic health care record system across the whole cancer pathway.

**Objective:**

This study aims to establish the quality of cancer-related clinical coding in NCL primary care, encompassing both quantitative measures (eg, coding completeness and diversity) and qualitative dimensions such as clinical relevance and workflow alignment.

**Methods:**

This was a mixed methods QI project in which we combined an observational dataset review and qualitative data from stakeholder interviews, workshops, and discussions. In the dataset review, we evaluated completeness, diversity, validation, and granularity in cancer clinical coding along the patient cancer pathway, which was split into three domains: (1) patient characteristics and risk factors, (2) cancer screening attendance, and (3) living with cancer. It was conducted in NCL primary care electronic health record systems, covering a population of over 1.4 million adults across 5 boroughs.

**Results:**

Cancer-related clinical coding in NCL primary care revealed significant gaps despite high completeness for ethnicity (912,679/1,055,083, 86.5%) and language (898,023/1,307,601, 68.7%). Employment status (29,848/1,229,644, 2.4%) and family history of cancer (183,424/1,236,580, 14.8%) were underrecorded, with wide variation in coding practices. Screening data showed good alignment with national datasets for cervical and bowel screening but fragmented and inconsistent breast screening data due to a lack of standardized codes. Cancer diagnosis coding was incomplete (4604/5260, 87.5% recorded), and treatment and staging data were almost entirely absent, limiting proactive management of long-term consequences. Stakeholder input highlighted inconsistent template use, limited data updates, and insufficient incentives as key barriers to better coding.

**Conclusions:**

The QI project has provided a detailed insight into the many dimensions of cancer coding and sheds light on many factors that underpin variation and coding preference. We offer a number of recommendations. The prioritized ones include the need for a cancer clinical coding data framework for primary care supported by appropriate funding and incentivization; improvements in the breast screening pathway and its interface with primary care; improvements in the quality of secondary care information that is sent to primary care; and dissemination of the importance of coding of cancer activity in primary care.

## Introduction

### Overview

Clinical coding of cancer-related data in primary care supports accurate and timely data collection, analysis, and reporting of cancer diagnoses and treatments, which in turn facilitates high-quality patient care [1,2]. Consequently, incomplete or inaccurate clinical coding of cancer-related data has significant implications across the cancer pathway. For example, cancer prevention efforts, including information provision, vaccination, and screening, may be restricted if it is not possible to identify eligible individuals based on data available in primary care records (eg, age, sex, health behaviors, previous medical history) and follow up with those who have not engaged (eg, those who have not responded to previous cancer screening invitations) [3]. Missing data for cancer risk-factors, such as family history or previous medical history, may undermine appropriate referral of symptomatic patients for cancer investigation [4]. Similarly, missing data on precancerous conditions such as Barrett’s esophagus or bowel polyps may prevent health care professionals from providing information and support to patients about managing their condition and personal risk and result in patients being excluded from relevant safety-netting efforts and surveillance pathways [5]. Meanwhile, the underreporting of cancer cases can lead to an underestimation of the true cancer burden within a population and limit the ability of primary care to support patients during cancer diagnosis and treatment [5,6]. Beyond clinical impact, poor coding may also contribute to financial losses for health care providers and hinder effective service planning at both practice and system levels [7].

Primary care cancer coding data are often variable and suboptimal, with a poor evidence base for improvement [8-10]. A systematic review by Thiru et al [11], concluded on the lack of standardized measures for data quality, which is supported by previous studies showing the heterogeneity of quality assessment methods in primary care coding [11,12]. Thiru et al [11], uncovered studies that assessed primary electronic patient record data and studies that reviewed survey and questionnaire data. They also found that data quality (reliability) was usually measured with rate comparisons and data validity was expressed under a range of terms (completeness, correctness, accuracy, consistency, and appropriateness), which were rarely defined [11].

Previous studies have identified the need for better communication about patients who have been diagnosed with cancer between primary and secondary care [13], and a UK study reported that 1 in 5 patients with cancer were not recorded to have a cancer diagnosis in primary care records [6]. In England, relatively robust audit systems and regulatory oversight exist to regulate coding for cancer diagnosis, cancer interventions, and procedures in secondary care, underpinned by financial incentives [14,15]. In contrast, equivalent governance mechanisms do not exist in primary care. Clinical entries in primary care rely on SNOMED CT (Systematized Nomenclature of Medicine – Clinical Terms), but there are no national standards or frameworks specifically guiding cancer coding across the whole pathway. Instead, coding behavior is shaped primarily by the Quality Outcomes Framework (QOF) [16], which provides financial incentives for documenting selected conditions and activities. For cancer, QOF incentivizes the coding of a cancer diagnosis and the completion of cancer care reviews (CCRs) following diagnosis—although CCRs were removed from general practitioner (GP) QOF contracts in April 2025 [16,17]. Importantly, QOF does not cover the full cancer pathway. Moreover, primary care coding systems often encourage diversity rather than consistency, such as having multiple codes to describe identical clinical events [10,18] (eg, “smoker,” “cigarette smoker,” “moderate cigarette smoker”). Lack of regulation and research evidence about cancer coding consistency and variation restricts the development of targeted quality improvement (QI) measures. Consequently, there is a need to understand how the quality of clinical coding data in primary care varies to influence outcomes across the cancer pathway. This holistic approach can inform suitable interventions for optimizing the quality of primary care coded data to deliver valuable improvements in cancer prevention, referral, diagnosis, and treatment outcomes [13].

### Cancer Coding and Data Curation

The process of how patient information is recorded, updated, and monitored in electronic health records is sometimes called “data curation.” Primary and secondary care data curation is influenced by multiple drivers, which in turn affect data quality. In the United Kingdom, the National Cancer Registration and Analysis Service (NCRAS) [19] provides guidance and support for clinical coding of cancer diagnoses and treatment in acute care. NCRAS has developed a set of coding standards that are incorporated in nationally commissioned datasets such as the Cancer Outcomes and Services Dataset and Systemic Anti-Cancer Therapy dataset, which provide guidance on the coding of cancer diagnoses and treatments. The National Health Service (NHS) primary care coding system is called SNOMED CT [20]. The SNOMED CT does not have standard clinical coding specifications for cancer data.

Acute and mental health trusts have standard procedures for regular quality inspections of their coded clinical data for inpatient and day-case episodes by approved clinical coding auditors, who aim to demonstrate compliance with national clinical coding standards [21]. The regulatory component is important, as it supports high-quality data collection that supports secondary uses of the data, such as collaborations with academic and research departments (eg, the “Getting It Right First Time” program to reduce unwarranted variation) [22]. There is no equivalent process for auditing the assignment of the terminology SNOMED CT, which is used in primary care.

Reasons for primary care coding incompleteness and inconsistencies are well documented and include time pressures, finding the right code, and motivation to code [12,23]. As part of the development work that led to this study, NCL Cancer Alliance conducted an online survey of GP respondents in London to understand barriers to good-quality clinical coding in primary care. The findings included lack of standardized coding practices, scarcity of dedicated staff time to code, and inadequate training around coding (for the full list of barriers see Table S2 in Multimedia Appendix 1 [24]).

### Context for the Quality Improvement Project

The aim of this QI project was to assess the completeness and variation of clinical cancer coding in NCL primary care data and to understand the reasons for this. The output of this QI project was to develop a robust “case for change” [25] for relevant improvement solutions. The long-term goal of the project was to improve service planning, pathway delivery, and redress health inequalities. The QI project was carried out by the NCL Cancer Alliance team with support from researchers at the Queen Mary University of London.

Our objectives were to: first, examine the quality of clinical coding of cancer-relevant risk factors, processes, and outcomes, encompassing both quantitative measures (eg, coding completeness and diversity) and qualitative stakeholder perspectives (eg, barriers, enablers, clinical relevance, and workflow alignment); second, use data from the first objective to develop recommendations to support data improvement in primary care.

## Methods

### Overview

This was a mixed methods QI project combining an observational dataset review and stakeholder interviews, workshops, and discussions. The observational dataset and analysis were based on data searches designed in EMIS (Egton Medical Information Systems) Web by Enfield GP Federation. These were set up to take a snapshot of information held in GP records as it stood on October 31, 2023, so that results are consistent and comparable across practices. All participating GP federation teams then ran the searches between November 2023 and January 2024. The results were shared with the NCL Cancer Alliance, which completed the data analysis in April 2024. Preliminary insights from the early stages of qualitative data collection (eg, advice from group interviews) informed the quantitative analysis (eg, process barriers in breast screening to ethnicity codes recorded in 2 different parts of EMIS Web) and the development of the workshop themes and questions. The formal qualitative analysis was conducted after all qualitative data had been collected; this section of analysis was carried from October to December 2024 (Figure S1 in Multimedia Appendix 1 depicts the project timeline).

### Setting and Participants

We gathered data on clinical coding from NCL primary care GP systems covering an adult population of >1.4 million. The qualitative data included email conversations, 2 semistructured interviews, and 2 workshops with key primary care stakeholders. The qualitative methods and analyses are reported according to the COREQ (Consolidated Criteria for Reporting Qualitative Research) checklist (Table S3 in Multimedia Appendix 1) [24].

### Data Collection

We extracted GP data from the electronic health care system, EMIS Web [26], the sole GP electronic health care record provider for all GP practices in NCL. Data were obtained using built-in “searches” within EMIS Web. These are configurable protocols designed to retrieve coded patient information based on predefined clinical or demographic criteria, referred to as data domains (eg, ethnicity, smoking status, cancer diagnosis, or treatment history). Each search identifies patients meeting the selected criteria based on structured clinical codes. The output of these searches is presented in the form of “reports,” which summarize the number of patients meeting each criterion and can be exported for further analysis. Figure S2 in Multimedia Appendix 1 provides an example of a search. We used the SNOMED CT [20,27] and EMIS Web clinical codes [28], which coexist in patients’ records [29]. The NCL GP federations performed the searches; a GP federation is a group of general practices working collaboratively as an organizational entity to improve patient care, share resources, and enhance service provision within the local health economy [30].

Table 1 illustrates the number of GP practices across each GP federation or primary care network (PCN) in NCL and the completeness of report returns across the 26 searches that were built and run. A PCN in Islington that was not part of the Islington GP Federation (Islington North 2) did not participate in this project. A partial return is where some GP practices have generated results in a search and some have not due to there either being no patients who meet the criteria or technical constraints that prohibit the search from running for particular practices.

**Table 1:** Shows the number of general practitioner (GP) practices within each GP federation or primary care network (PCN) and the completeness of the reports that were requested.

Category		GP entity						
		Barnet^a^, n	Camden^a^, n	Camden Health Evolution^a^, n	Enfield^a^, n	Haringey^a^, n	Islington^a^, n	Islington North 2^b^, n
Number of GP practices		48	23	9	30	34	23	8
**Report status**								
	Complete	7	21	21	13	21	21	0
	Partial	17	5	5	13	4	4	0
	Did not return	2	0	0	0	0	0	26
	Search generated null results	0	0	0	0	1	1	0

^a^Represents GP federations.

^b^Represents a PCN.

Three personalized cancer care metrics—cancer care plan given, end-of-treatment summary, and holistic needs assessment—were unintentionally omitted from the original data request. This gap was identified by the NCL Cancer Alliance after data had already been submitted by all boroughs. To partially address this, Enfield Federation, which had local access, conducted the relevant searches for its own borough (covering the 12 months up to October 2023). Due to timing and resource constraints, it was not feasible to repeat this process across the other boroughs. Enfield’s data were compared with HealtheIntent cancer registries [31], which span the entire NCL adult population; while full coding quality could not be assessed, we were able to examine the relative frequency of these codes across populations.

### Data Quality Assessment Method

We followed the approach taken by Pineda-Moncusi et al [32], who examined ethnicity data at a large scale and defined data quality across completeness, coverage, and granularity (most prevalent clinical codes used).

We drew on existing cancer data frameworks [33,34] to conceptualize different cancer pathway stages and identify relevant cancer codes. Within cancer alliances, there are established programs [35] spanning the entire pathway—from awareness and prevention through to living with and beyond cancer. Our aim in this study was to create a comprehensive and holistic dataset to review, thereby informing future recommendations. To do this, we built on this existing knowledge, incorporated earlier work from our team [36], and collaborated with our wider NCL Cancer Alliance team to scope data items across each pathway stage. Although we recognized that some of these items were unlikely to be routinely coded in primary care, there was no empirical evidence to confirm this; therefore, part of the purpose of this QI project was to assess the current state of coding. End-of-life care was deemed out of scope for this work. The Enfield GP Federation digital team built EMIS Web searches to cover each element of the pathway and shared these with other NCL GP federations’ IT teams to run for each borough. Enfield GP Federation’s IT team oversaw the communication, search development, search dissemination, and data submissions. Raw data were transferred to the NCL Cancer Alliance.

The data protection officer for NCL primary care assured that all data sharing complied with UK General Data Protection Regulation.

### Qualitative Data Collection

A convenience sample of key stakeholders with expertise in clinical coding were invited (over email and through a GP bulletin) to attend an online group workshop through existing contacts (including GPs, project and program managers, IT staff, and academic researchers). They were told that they were being invited to discuss the quantitative findings relating to clinical coding. We conducted 2 semistructured group interviews and 2 workshops with 11 primary care stakeholders. We also reviewed email correspondence from the Enfield GP Federation team, which captured responses to queries arising from the initial round of quantitative data analysis. These communications provided a systematic method for clarifying data gaps and process-related issues and directly informed the development of key questions explored during the subsequent stakeholder workshops.

All interviews and workshops were held remotely over Microsoft Teams between April and August 2024 (Table S4 in Multimedia Appendix 1) and conducted by AB (female, GP clinical lead for Innovation and Integration at NCL Cancer Alliance). Sessions were also facilitated by 2 other members of the research team, GR (male, head of Data and Analytics, NCL Cancer Alliance) and KT (female, senior innovation consultant, NCL Cancer Alliance). Semistructured interview and workshop topic guides were developed by AB, KT, and GR. We presented key findings for discussion. All remote sessions were video recorded and transcribed using the Microsoft Teams record and transcription functions. Transcripts were not shared with stakeholders.

### Analysis

Quantitative data were analyzed descriptively across the different boroughs to characterize coding patterns. In total, 26 searches were built and run. We analyzed these data domains: patient ethnicity, main language spoken, weight or BMI, alcohol consumption, smoking status, family history of cancer, employment status, environmental pollutants exposure, carer, cancer screening attendance, cancer fast-track referrals, presence of malignant neoplastic disease, treatment regimen, and attendance at CCR. To ensure transparency and facilitate replication as far as possible, we have included these extracted search terms in Textbox S1 in Multimedia Appendix 1.

Codes were descriptively analyzed for the following features (1) coding completeness, (2) coding diversity, (3) data validation, and (4) granularity of coding (Table 2).

**Table 2:** Definitions and methods used to assess clinical coding quality and completeness.

Definitions	Methods
Coding completeness^a^	Percentage of eligible patients with a relevant SNOMED CT (Systematized Nomenclature of Medicine – Clinical Terms) or EMIS (Egton Medical Information Systems) clinical code captured. This reflects the presence or absence of a code rather than the true or expected prevalence of the underlying condition.
Coding diversity^b^	Number of unique SNOMED CT or EMIS codes used to describe the same information (eg, patient’s weight). This serves as a practical proxy for coding breadth, acknowledging limitations such as inaccessible child codes and variation in code list size.
Data validation	Comparison of coding completeness in EMIS Web with national or local reference datasets to assess missed or uncoded data. Calculated as EMIS Web completeness divided by completeness reported in the comparator dataset for the same coded item. Ratios may exceed 100% if EMIS Web demonstrates higher apparent completeness. Comparator datasets included HealtheIntent, Cancer Waiting Times, the National Cancer Registry, South East London Cancer Alliance data^c^, and the NHS Futures Screening Dashboard.
Granularity of coding	Review of the most frequently used codes to assess specificity, for example the use of general codes such as “current smoker.”

^a^Coding completeness describes the proportion of eligible patients in the denominator population who have a relevant code captured in their primary care record. This definition is intended to reflect the presence or absence of a code rather than to imply that the observed percentages represent the true or expected prevalence of the underlying clinical characteristic or diagnosis. For example, a coding completeness of 0.3% for malignant neoplastic disease reflects the proportion of the registered population with a relevant cancer code, not an assessment of whether this proportion is “correct” or “incorrect.” To distinguish between the technical measurement of code capture and the interpretive question of whether coding levels are as expected, we also applied the concept of coding validation. Coding validation involves comparing observed coding completeness against external standards or datasets (eg, cancer wait times) to assess whether the recorded levels are appropriate and consistent with known population rates. In this way, completeness provides a descriptive measure of the presence of codes in primary care data, while validation enables assessment of their adequacy and alignment with clinical or epidemiological expectations.

^b^Coding diversity was defined as the number of unique codes captured in EMIS Web for each search. We recognize this is a practical proxy rather than a full measure, as EMIS Web does not easily expose all child codes, and many codes (eg, for rare conditions or languages) will naturally yield zero results. Some code lists are also inherently larger than others. A distinct count of unique codes therefore provides useful context on the breadth of coding options observed while acknowledging these limitations.

^c^South East London Integrated Care Board (ICB) and Cancer Alliance has its own population health dashboard that overlaps with the data and definitions in this study, meaning it is valid for comparison where we have no other published source. This dashboard is not publicly available.

Data completeness and validation analysis was carried out against all submitting GP practices’ adult populations as of January 2024 (Table S5 in Multimedia Appendix 1). Practice populations are relatively stable month to month, so comparing October 2023 search results with the adult population in January 2024 is considered valid. Practices that did not submit data for a profile were excluded from the analysis to maximize data integrity (Table S6 in Multimedia Appendix 1).

Table 3 lays out the report names across the time frames for which the data was searched for, the denominator population, and the validation database used for comparison. Additionally: (1) data on body weight and BMI were assessed through a combined height and weight search); (2) smoking status was assessed through three separate searches; (3) family history of cancer was assessed based on any recorded code, rather than limiting analysis to the preceding 24 months (as the GP federations’ IT team were aware this would be captured at one point in records); and (4) breast screening data were retrieved through four distinct searches: screening attendance, normal results, abnormal results, and cancer detected.

**Table 3:** Each report name shown against time period covered for each report, the denominator population, and the validation database used for comparison, including the validator time period.

Report name	Time period	Denominator population characteristics (age in years)	Validator database	Validator metric and time period
Ethnic origin	Recorded ever	>18	HealtheIntent	Ethnicity coding (April 2024)
Main language spoken	Recorded ever	>18	HealtheIntent	Main language spoken (April 2024)
Employment status	October 2021-October 2023	>18	No comparator	—^a^
Weight or BMI recorded	October 2021-October 2023	>18	South East London Cancer Alliance data completeness	Weight or BMI recorded (April 2024)
Current smoker (recorded in last 24 months)	October 2021-October 2023	>18	HealtheIntent	Current smoker status (April 2024)
Any smoking status (recorded in last 24 months)	October 2021-October 2023	>18	South East London Cancer Alliance data completeness	Any smoking status (April 2024)
Alcohol consumption record	October 2021-October 2023	>18	South East London Cancer Alliance data completeness	Alcohol consumption record (April 2024)
Family history of neoplasm	Recorded ever	>18	No comparator	—
Environmental pollutants	October 2021-October 2023	>18	No comparator	—
Fast track referral coding	New episode added in last 12 months	>18	Cancer Wait Times	Urgent suspected cancer referrals (2023)
Cancer–bowel: did not return screening kit	October 2023-2 years 6 months	60-74	No comparator	—
Cancer–bowel: screening uptake	October 2023	60-74	NHS Futures Screening Dashboard	Bowel screening uptake August 2023 (50-70 years)
Cancer–bowel: abnormal result	October 2023-2 years 6 months	60-74	No comparator	—
Cancer–breast: screened	October 2023-3 years 6 months	50-70 (female)	NHS Futures Screening Dashboard	Breast screening uptake August 2023 (50-70 years)
Cancer–breast: abnormal result	October 2023-3 years 6 months	50-70 (female)	No comparator	—
Cancer–breast: normal result	October 2023-3 years 6 months	50-70 (female)	No comparator	—
Cancer–breast cancer detected	October 2023-3 years 6 months	After screened for breast cancer (female)	National Cancer Registry	Breast cancer diagnosis via screening route February 2020 to July 2023 (3.5 years)
Cancer–cervical: adequate smear	October 2023-3 years 6 months	25-49 (female)	NHS Futures Screening Dashboard	Cervical screening uptake December 2023 (25-49 years)
Cancer–cervical: adequate smear	October 2023-5 years 6 months	50-64 (female)	NHS Futures Screening Dashboard	Cervical screening uptake December 2023 (50-64 years)
Malignant neoplastic disease	New episode added in last 12 months (as of October 2023)	>18	National Cancer Registry	Rapid Cancer Registration – New Diagnosis (2023)
Malignancy stage	New episode added in last 12 months (as of October 2023)	>18	National Cancer Registry	Rapid Cancer Registration – New Diagnosis (2023)
Treatment regimen	New episode added in last 12 months (as of October 2023)	>18	Cancer Waiting Times	Treatment starts (2023)
Cancer care review	New episode added in last 12 months (as of October 2023)	Cancer-diagnosed patients who had a care review	No comparator	—
Has a carer	Recorded ever	>18	No comparator	—

^a^Not applicable.

### Missing Data

There were challenges with data collection in participating practices. Running searches at scale across several practices was technically difficult at times. These searches would often time out and fail, resulting in incomplete data collection. Despite repeated running of these searches to attempt to get a complete dataset, failures persisted (Table S3 in Multimedia Appendix 1 and its linked notes provide more detail on this process).

Barnet had the highest level of missing data across the 26 reports, although data gaps were present in all boroughs. The ethnic diversity of Barnet is broadly comparable to the rest of NCL, and given that data from Barnet were available for other nonethnicity reports, we do not believe these gaps materially skew the overall findings. Table S7 in Multimedia Appendix 1 illustrates the proportions of practices that submitted data across each of the 26 searches.

**Figure 1:**
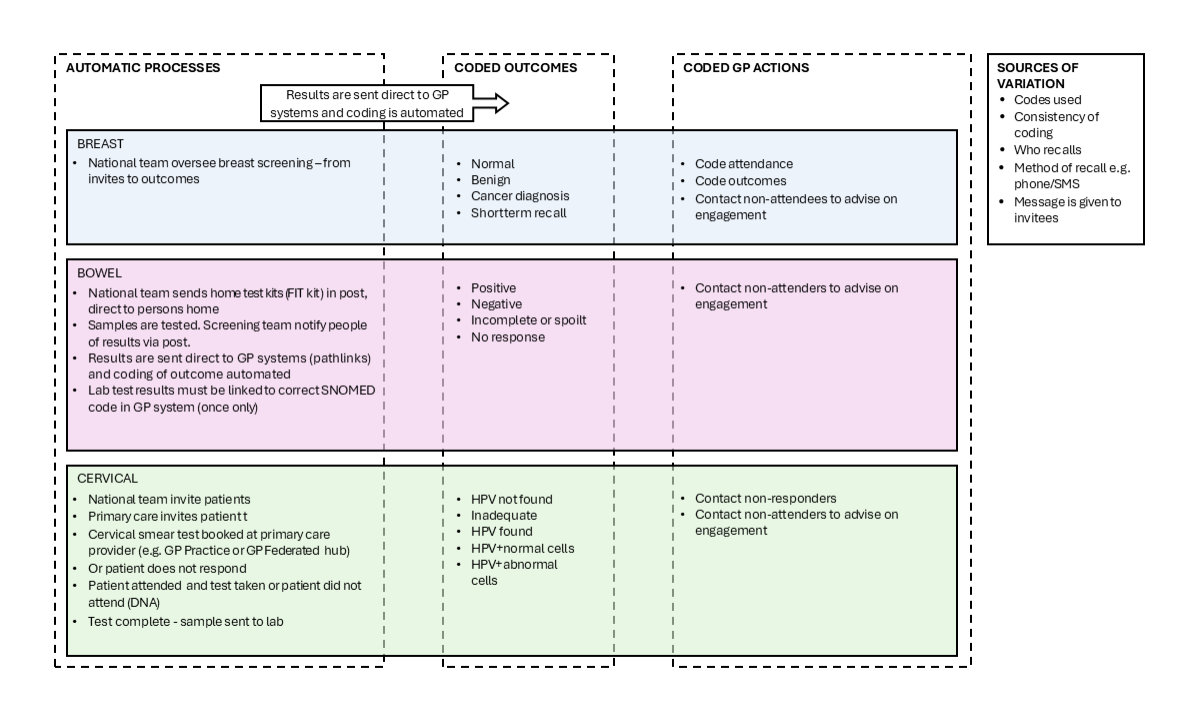
Diagram showing how codes are generated for all cancer screening programs in primary care.

The searches did not examine comparable data across each screening pathway. Direct comparisons between breast, colorectal, and cervical screening were not feasible, as primary care is not uniformly responsible for screening data across the 3 screening pathways. As a result, a process mapping exercise was conducted to trace the pathways of each screening program into primary care, identifying recordable actions within primary care settings as shown in Figure 1.

The qualitative data were analyzed by SB, a female behavioral science PhD student, and GB, a female applied health researcher. Both had previous experience conducting qualitative research. The analysis began in October 2024, after qualitative data collection had finished. SB listened to all video recordings and checked the transcripts for accuracy to become familiar with the study context and dataset before coding the data.

Qualitative data collection aimed to contextualize and validate quantitative findings rather than to achieve theoretical saturation. As this study adopted a QI focus, data collection was concluded once sufficient breadth of perspectives had been obtained and no new issues emerged that were relevant to the study objectives.

The analysis was conducted in Google Sheets using framework analysis [37]. Key excerpts from transcripts, email conversations, and comments posted in the chat during the workshops were copied into Google Sheets. All qualitative data were analyzed together. Raw data were arranged into columns. Each column represented a single code, and each row included raw data (eg, a verbatim quote labeled by data source and speaker) pertaining to that code.

An initial framework of 3 themes was developed deductively based on the findings of the quantitative analysis. SB coded the data into each theme before arranging the data into subthemes inductively. SB and GB met after 10%, 50%, and 100% of the data had been coded to discuss and revise the coding framework. A reflexive diary was kept and referred to throughout the analysis, which included field notes and impressions recorded by the researcher who conducted the interviews and workshops (AB) and the researchers who conducted the analysis (SB and GB). Regular meetings were held with the research team to clarify contextual details and discuss key interpretations. A reflexivity statement is available in Textbox S2 in Multimedia Appendix 1.

### Ethical Considerations

This study was designed as a QI and health service enhancement initiative and therefore ethics approval was not applied for.

All data analyzed were fully deidentified at source, aggregated, and subject to small-number suppression (<5) in accordance with local information governance standards to protect patient confidentiality. The dataset was generated through local GP federations, with each federation running standardized searches and submitting aggregated data to Enfield Federation. Enfield Federation then securely transferred the data to the NCL Cancer Alliance for analysis.

The NCL primary care data protection officer reviewed and assured all data-sharing processes, confirming they aligned with the UK General Data Protection Regulation and the Caldicott Principles. A Data Protection Impact Assessment was not required, as the data used contained no identifiable information and presented no privacy risk.

## Results

### Principal Findings

We present our findings combining our descriptive analyses of clinical codes (quantitative findings) and the factors influencing coding (qualitative findings), in three themes: theme 1, precancer pathway, which includes codes relating to demographic characteristics, physical characteristics, risk factors (eg, family history), and cancer referrals; theme 2, cancer screening, which includes codes relating to screening invitations and uptake; and theme 3, postcancer diagnosis, which includes codes relating to staging, treatment, primary care surveillance, and follow-up.

In Table S8 in Multimedia Appendix 1, we include further details on data items included for each theme. Key findings are summarized for each theme before the results of the quantitative and qualitative analyses are presented.

### Theme 1: Precancer Pathway

#### Overview

Completeness, code diversity, and validation of precancer codes are presented in Table 4 (Table S9 in Multimedia Appendix 1 provides additional detail on granularity).

**Table 4:** Descriptive analyses for data domains in theme 1 (precancer), showing completeness, code diversity, and validation of codes that were searched for in this theme.

Report name	Time period	Completeness and code diversity		Code validation	
		Completeness (% eligible coded), n/N (%)	Number of unique SNOMED CT^a^ or EMIS^b^ codes, n	Comparator source	EMIS completeness vs comparator, n/N (%)
Ethnic origin (coding)	Recorded ever	912,679/1,055,083 (86.5)	375	HealtheIntent	86.5/90.7 (95.4)
Main language spoken	Recorded ever	898,023/1,307,601 (68.7)	338	HealtheIntent	68.7/62.8 (109.4)
Employment status	24 months up to October 2023	29,848/1,229,644 (2.4)	87	No comparator for coding prevalence of this data item sourced	No comparator for coding prevalence of this data item sourced
Weight or BMI recorded	24 months up to October 2023	485,660/1,159,241 (41.9)	34	SELCA^c^	41.9/45.8 (91.5)
Current smoker (recorded in last 24 months)	24 months up to October 2023	90,029/1,236,580 (7.3)	47	HealtheIntent	7.3/14.9 (49)
Any smoking status (recorded in last 24 months)	24 months up to October 2023	530,720/1,185,812 (44.8)	117	SELCA	44.8/48.6 (92.3)
Alcohol consumption record	24 months up to October 2023	222,753/1,236,580 (18)	39	SELCA	18/29.2 (61.6)
Family history of neoplasm	Recorded ever	183,424/1,236,580 (14.8)	479	No comparator for coding prevalence of this data item sourced	No comparator for coding prevalence of this data item sourced
Environmental pollutants	24 months up to October 2023	494/1,113,575 (0.04)	45	No comparator for coding prevalence of this data item sourced	No comparator for coding prevalence of this data item sourced
Fast track referral coding	New episode added last 12 months up to October 2023	61,562/1,506,746 (4.1)	34	Cancer Waiting Times	61,562/78,989 (78)

^a^SNOMED CT: Systematized Nomenclature of Medicine – Clinical Terms.

^b^EMIS: Egton Medical Information Systems.

^c^SELCA: South East London Cancer Alliance.

The completeness of ethnicity coding was high, with 86.5% (912,679/1,055,083) of records containing an ethnicity code. A total of 375 distinct SNOMED CT codes were identified, with a 15.8% variation in coding completeness across boroughs. “Other White background - ethnic category 2001 census” made up 18.6% (170,190/912,679) of total codes captured in NCL. Language coding completeness was 68.7% (898,023/1,307,601) across the eligible population, which compares favorably with the comparator database coverage of 62.8% (929,987/1,482,024). A total of 338 unique codes were identified, with borough-level variation of 15.2% (Camden=75.3% and Enfield=60.1%). The top 2 most prevalent codes for language were “Main spoken language English” (527,227/898,023, 58.7%) and “Main spoken language NOS” (48,311/898,023, 5.4%). Employment status coding was minimal, with completeness at only 2.4% (29,848/1,229,644). The most frequently recorded codes related to unemployment (11,801/29,848, 39.5%) and work-related stress (5063/29,848, 17%), with a total of 87 distinct codes identified.

Coding completeness for BMI was 41.9% (485,660/1,159,241), with 34 individual codes used to describe weight and BMI. The SNOMED CT code for BMI accounted for 88.7% (430,736/485,660) of recorded entries. Overall, 44.8% (530,720/1,185,812) of the population had a recorded smoking status, including classifications such as current smoker and ex-smoker. Approximately 170 variations of smoking-related codes were identified. Data validation demonstrated near completion. Alcohol consumption was recorded in 18% (222,753/1,236,580) of patient records, with borough-level variation ranging from 13.9% (39,198/281,807) to 21% (64,255/281,807). A total of 39 different codes were identified. The “AUDIT-C” screening tool, which assesses excess alcohol consumption, was the prevalent code at 43.4% (96,619/222,753).

Family history of cancer across NCL showed that 14.8% (183,424/1,236,580) of records contained relevant codes, with 479 unique codes identified. There was no available comparator dataset for this parameter. The 2 most prevalent codes were “FH-Cancer” and “FH-Neoplasm” at around 17% each. Coding of environmental exposure was extremely limited, with a completeness rate of 0.04% (494/1,113,575), rendering the data unsuitable for analysis.

The incidence of suspected cancer referrals among eligible patients was 4.1% (61,562/1,506,746), with a 1% variation between the highest and lowest referring boroughs. The comparator database indicated a referral rate of 5.2% (78,989/1,506,746) for NCL residents. The analysis excluded deceased, temporary, and deregistered patients. The discrepancy between the study findings and comparator data was largely attributable to deceased patients.

#### Factors Influencing Clinical Coding of Precancer Data in Primary Care

Stakeholders reported 3 key factors that contribute to the quality and completeness of clinical coding of precancer data in primary care: opportunities to collect or update precancer data, motivations and capacity to collect and code precancer data, and the nature of the systems used to code precancer data.

#### Opportunities to Collect or Update Precancer Data

Many stakeholders reported that a key opportunity to capture precancer data is through registration forms and health check appointments. Some explained that registration templates and the commissioning of health check appointments vary by practice, leading to inconsistencies. There was agreement that standardized templates could improve this, although only for those registering subsequently, and it was noted that patients may not provide information if questions are not mandatory and the purpose of data collection is not transparent. While a few GPs suggested that mandating questions could improve data completeness, others were concerned that this could introduce barriers to registration. Family history is not routinely coded but may be documented in free text within clinical notes or referral forms. Environmental exposure data is not routinely collected or standardized within primary care records.

Some stakeholders advised that a lack of data completeness for information that changes over time, such as main language spoken or smoking status, is due to limited opportunities to update patient data after registration unless patients schedule appointments with primary care (eg, long-term conditions review, new medication appointments, e-consultations). It was suggested this could be improved by offering annual health check appointments, inviting patients to update their information, and the sharing of information collected in secondary care.

#### Motivations and Capacity to Collect and Code Precancer Data

Many GPs indicated that local and national financial incentive schemes (such as the QOF [38]) influence whether they collect and code precancer data at registration and during patient consultations. Some reported that variation in coding completeness between boroughs could be attributed to differences in locally commissioned services. There was agreement that clinical coding is demanding of staff time and capacity, and that improvements to the quality and completeness of clinical coding are unlikely to continue beyond the period of incentivization.

There was a general consensus that, in the absence of mandates and incentives, GPs are motivated to code precancer data that are relevant to the clinical workflow, such as arranging appointments (eg, need for interpreter, has a carer), assessing eligibility for local-level services (eg, smoking cessation, vaccination, information provision), or management of a patient’s symptoms or a long-term condition. Some GPs admitted that they prefer to document precancer data in free text responses. This prevented interrupting the flow of conversation with patients, and, if needed, they could provide detail on more complex factors (eg, family history and exposure to environmental pollutants).

#### Nature of Systems Used to Code Precancer Data

GPs advised that coding will vary depending on whether patients register at GP practices using a paper or online form, whether registration data are coded into the system manually by staff or automatically, and which additional registration processing software practices have access to. It was also raised that there are multiple places within the system for data to be recorded. Furthermore, it was reported that some urgent suspected cancer referral forms may be available in the system but not trigger a SNOMED CT code. There was consensus that automated registration and data capture would improve the consistency and completeness of precancer data in primary care systems.

**Figure 2:**
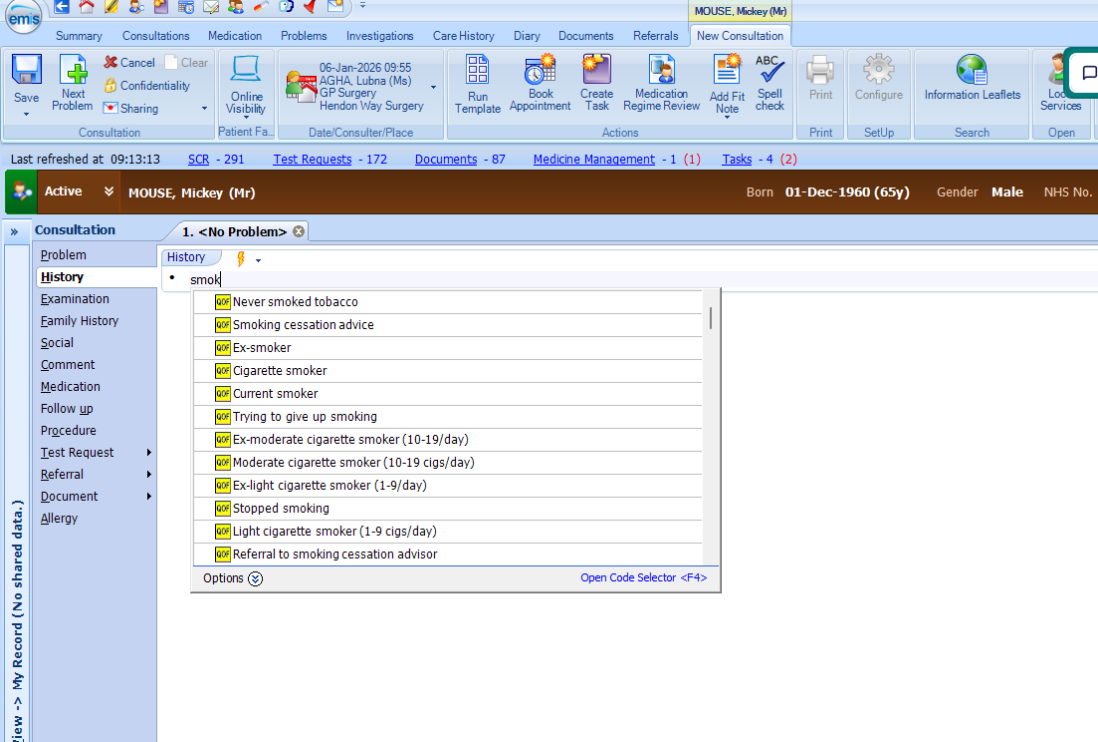
Screenshot of SNOMED CT (Systematized Nomenclature of Medicine – Clinical Terms) code options for term “smok” when typed into EMIS (Egton Medical Information Systems) Web, showing a drop-down menu of multiple code options that start with “smok.”.

Several GPs suggested that the consistency of coding is made challenging by the array of codes available for specific types of precancer data such as smoking status, BMI, and family history, where there are different levels and layers, codes with similar or ambiguous meanings, and historic codes and prompts that cannot be removed (Figure 2). GPs admitted that codes higher up the list or those labelled with QOF prompts are most likely to be selected, and a program manager advised that coding prompts should be reserved for data that are most important to capture.

### Theme 2: Screening Pathway (Breast, Bowel, and Cervical Screening)

Completeness, code diversity, and validation of cancer screening data is presented in Table 5 (Table S10 in Multimedia Appendix 1 provides additional detail on granularity).

**Table 5:** Descriptive analyses for data domains in Theme 2 (screening), showing completeness, code diversity, and validation of codes that were searched for in this theme.

Report name	Time period	Completeness and code diversity		Code validation	
		Completeness (% of eligible patients with code captured), n/N (%)	Number of unique SNOMED CT^a^ or EMIS^b^ codes in search, n	Data validation comparator source	EMIS data completeness as a proportion of comparator, n/N (%)
Cancer–bowel (aged 60-74 years; did not return screening kit)	Run date (October 2023)-2 years 6 months	65,762/188,939 (34.6)	4	No comparator for coding prevalence for kit DNRs^c^, only uptake and coverage	No comparator for coding prevalence for kit DNRs, only uptake and coverage
Cancer–bowel (aged 60-74 years; screened)	Run date (October 2023)-2 years 6 months	112,939/184,323 (61.3)	10	NHS^e^ Futures Screening Dashboard	61.3/62.2 (98.6)
Cancer–bowel abnormal result	Run date (October 2023)-2 years 6 months	2308/184,323 (1.3)	5	No comparator for coding prevalence for abnormal screening results, only uptake and coverage	No comparator for coding prevalence for abnormal screening results, only uptake and coverage
Cancer–breast (aged 50-70 years; screened)	Run date (October 2023)-3 years 6 months	79,321/175,986 (45.1)	30	NHS Futures Screening Dashboard	45.1/57.2 (78.8)
Cancer–breast (aged 50-70 years; abnormal result)	Run date (October 2023)-3 years 6 months	1646/114,606 (1.4)	7	No comparator for coding prevalence for abnormal screening results, only uptake and coverage	No comparator for coding prevalence for abnormal screening results, only uptake and coverage
Cancer–breast (aged 50-70 years; normal result)	Run date (October 2023)-3 years 6 months	71,043/165,126 (43)	3	No comparator for coding prevalence for normal screening results, only uptake and coverage	No comparator for coding prevalence for normal screening results, only uptake and coverage
Cancer–breast cancer detected	Run date (October 2023)-3 years 6 months	913/167,315 (0.5)	35	National Cancer Registry	913/731 (124.9)
Cancer–cervical (aged 25-49 years; adequate smear)	Run date (October 2023)-3 years 6 months	205,730/356,955 57.6%	103	NHS Futures Screening Dashboard	57.6/57.7 (99.8)
Cancer–cervical (aged 50-64 years; adequate smear)	Run date (October 2023)-5 years 6 months	97,233/139,233 (69.8)	98	NHS Futures Screening Dashboard	69.8/71 (98.3)

^a^SNOMED CT: Systematized Nomenclature of Medicine – Clinical Terms.

^b^EMIS: Egton Medical Information Systems.

^c^DNR: did not return.

^d^NHS: National Health Service.

#### Breast Screening

Coding for breast cancer screening uptake was recorded in 45.1% (79,321/175,986) of screening-eligible patients, with a 12% deficit compared with the comparator database. A total of 30 unique codes were used to document breast screening activity. The number of breast cancer diagnoses following abnormal results exceeded those in the comparative dataset. The most frequently used code was “Mammography normal,” accounting for 39.2% (31,109/79,321) of coded entries. The qualitative data (semistructured interviews and workshops) verified much of the persistent quality issues in the breast screening service, particularly its interface with primary care. This data also highlighted problems such as significant delays in breast screening attendance and nonattendance notifications; delivery of notifications by letters that contain multiple patients on a single sheet and therefore require manual separation; and counterintuitive patient reminders. For instance, proactive reminders for women upon turning age 50 years can cause confusion, because screening invitations may not be issued until age 53 years, with no flexibility for earlier appointments.

#### Bowel Cancer Screening

Codes for bowel cancer screening suggested an uptake of 61.3% (112,939/184,323). The proportion of screening-eligible patients who did not return test kits was 34.6% (65,762/188,939). Coding of abnormal results was documented in 1.3% (2308/184,323) of cases. The fecal immunochemical test (FIT), which underpins bowel screening, was consistently coded as “Bowel cancer screening program faecal occult blood test” within EMIS Web. The most frequently used codes were for normal and abnormal fecal occult blood (FOB) test results. The qualitative data (semistructured interviews) showed us that the bowel screening processes and coding practices were clearly defined for patients who undergo screening (aligned to QOF-funded activity). However, variability exists in recall, reminder, and engagement activities for nonresponders. Those who did not participate in screening were often coded using a non-SNOMED CT term, “No response to BCSP invitation.”

#### Cervical Screening

Cervical screening data in the eligible population, coded as having had a smear, were 57.6% (205,730/356,955; in the 25 to 49 years age group) and 69.8% (97,233/139,233; in the 50 to 64 years age group). Both numbers aligned with the comparator database at over 99% (57.6%/57.7%) and 98% (69.8%/71%). A total of 98-103 individual codes were used to describe cervical screening coverage among the 2 eligible age cohorts. The qualitative data (semistructured interviews with GP federation’s IT team) revealed that cervical screening processes were also clearly defined and aligned with the funding route (QOF). Variability existed in the recall and engagement activities for nonresponders. Reports for cervical nonengagement and recall were not developed because it was understood that data would not exist.

#### Factors Influencing Clinical Coding of Cancer Screening Data in Primary Care

Stakeholders explained that the coding of cancer screening data in primary care is influenced by primary care staff motivations and the ease of coding screening results in the system.

#### Relevance of Coding Cancer Screening Data to Clinical Workflow

Primary care staff described varied practices in whether cancer screening attendance was recorded. While some mentioned manual efforts or automated systems for reminding patients about upcoming screening appointments or contacting patients when they were notified of nonattendance, many admitted that they did not code screening attendance and follow up for those who did not attend. In discussing reasons for this, GPs and program managers indicated that coding was motivated by mandates and incentives (QOF) that are often only short term.

Many GPs agreed that recording screening data or contacting those who did not attend was not clinically relevant to primary care workflow and believed it was under the remit of national teams that run the programs. A few also noted that they did not have the most up-to-date information to monitor and facilitate screening attendance as they had received incorrect system prompts around screening attendance and were not aware of changes to screening eligibility. While some GPs and program managers noted local-level efforts to support and improve cancer screening attendance, there was general consensus that this is dependent on practice capacity to follow up those patients and is challenging due to competing priorities.

#### Ease of Coding Screening Results in Primary Care

GPs and program managers raised that coding breast cancer screening data is time consuming and demanding because paper results are sent to primary care with 2 patients’ results per page, meaning they must be cut in 2 before being filed. Some recounted making requests for results to be sent electronically to streamline this process but had accepted that the system cannot be changed. GPs and program managers reported that the multitude of coding options for breast and cervical cancer screening results (eg, cervical screening, smear, cervical smear) contribute to coding inconsistencies. There was agreement that standardized coding could improve this. In contrast, stakeholders reflected that processing bowel cancer screening results is straightforward because the codes on the screening results letters are easy to match to those on the system (SNOMED CT). However, some highlighted that FOB codes (which relate to guaiac fecal occult blood testing [gFOBt] that is no longer used in the bowel screening program) [39] are still being used to code FIT screening results. Some reported that there is unified understanding in primary care that these legacy codes relate to FIT results and that incentive schemes for screening data still acknowledge these codes. However, one GP raised that legacy FOB codes may not be acknowledged in data searches for symptomatic FIT results.

### Theme 3: Postcancer Diagnosis

#### Overview

Completeness, code diversity, and validation for postcancer codes are presented in Table 6 and in Table S12 in Multimedia Appendix 1 (Table S11 in Multimedia Appendix 1 provides additional detail on granularity).

**Table 6:** Descriptive analyses for data domains in Theme 3 (postcancer diagnosis), showing completeness, code diversity, and validation of codes that were searched for in this theme.

Report name	Time period	Completeness and code diversity		Code validation	
		Completeness (% of eligible patients with code captured), n/N (%)	Number of unique SNOMED CT^a^ or EMIS^b^ codes in search	Data validation comparator source	EMIS data completeness as a proportion of comparator, n/N (%)
Malignant neoplastic disease	New episode added last 12 months up to October 2023	6,044/1,506,746 (0.4)	677	National Cancer Registry	4604/6319 (73)
Malignancy stage	New episode added last 12 months up to October 2023	6/64,977 (0.01)	4	National Cancer Registry	6/3536 (0.2)
Treatment regime (if coded)	New episode added last 12 months up to October 2023	315/942,260 (0.03)	35	Cancer Waiting Times	315/10,574 (3)
Cancer care review	New episode added last 12 months up to October 2023	3953/1,236,580 (0.3)	1	No comparator for coding prevalence of this data item sourced	No comparator for coding prevalence of this data item sourced
Has a carer	Recorded ever	14,513/1,229,644 (1.2)	17	No comparator for coding prevalence of this data item sourced	No comparator for coding prevalence of this data item sourced

^a^SNOMED CT: Systematized Nomenclature of Medicine – Clinical Terms.

^b^EMIS: Egton Medical Information Systems.

The proportion of newly diagnosed cancers coded in EMIS was 73% (4604/6319) of the expected figure. The EMIS searches excluded deceased patients; further data comparison suggests that deceased individuals could account for 11.8%-14% of new cancer diagnoses in the Rapid Cancer Registration Database, reducing the initial coding gap from 27% to approximately 13%.

Cancer staging data showed a total completeness of 0.2% (6/3536) when compared with the validation database. One borough recorded no staging codes. Cancer treatment coding showed a total completeness of 3% (315/10,574) when compared with the validator.

CCRs were coded for 66% (3953/5982) of eligible patients, aligning with the 73% (4604/6319) of new cancer diagnoses recorded in primary care. A single SNOMED CT code is used to document CCRs. A total of 1.2% (14,513/1,229,644) of patients aged 18 years and older had a recorded carer status. The code “Has a carer” accounted for 80.1% (11,631/14,513) of these entries. Beyond CCRs, other personalized cancer care quality indicator data are shown separately in Table S12 in Multimedia Appendix 1. Cancer care plans, end-of-treatment summaries, and holistic needs assessments were recorded in 1%-2% of patients with cancer across NCL.

#### Factors Influencing Clinical Coding of Postcancer Diagnosis Data in Primary Care

Stakeholders suggested that coding of patients’ cancer diagnoses and treatment data is influenced by the relevance of information to the clinical workflow in primary care, the quality of information sharing from secondary care, and the complexity and consistency of the systems used for coding.

##### Relevance of Coding Postcancer Diagnosis Data to Clinical Workflow

A few GPs mentioned that coding of cancer diagnoses will improve when incentivized through the QOF. However, some highlighted that coding information about treatment plans organized by secondary care is not perceived as relevant or a priority in daily practice. One GP also reflected that they sometimes felt reluctant to request information from patients during CCR appointments given that patients have already had to discuss their diagnoses in secondary care.

##### Quality of Postcancer Diagnosis Information Sharing

GPs suggested that cancer diagnosis information is not always shared by secondary care or that it may be sent with some delay. They also reported that information may be missing for patients who are diagnosed and treated privately or those who are diagnosed at an advanced stage whereby primary care is only notified of a cancer diagnosis through the receipt of postmortem information. When cancer diagnosis information is received, there is consensus among GPs that letters from secondary care are long and complex, meaning staff have to scrutinize the whole letter to find and extract the key information. Many agreed that diagnosis information and SNOMED CT codes that require coding should be placed at the top of these letters for easy translation into primary care records. Some GPs also lack trust in the accuracy of information received from secondary care due to finding previous errors in patient records. For this reason, a few expressed concerns about linked data between primary and secondary care if errors could not be redacted within primary care.

##### Consistency and Complexity of Systems Used for Coding Postcancer Diagnosis Data

GPs reported that there are multiple places within primary care–based systems (EMIS) where cancer information can be coded or entered as free text, as well as several different coding options that prevent consistent coding of cancer diagnoses. There was agreement that standardized templates were needed. GPs also reported that *ICD-10* (*International Statistical Classification of Diseases, Tenth Revision*) codes used in secondary care letters are not aligned to SNOMED CT codes used in primary care, which causes ambiguity. Additionally, some explained that practices vary in their capacity to ensure the quality of coding, such as whether formalized coding teams are used and given adequate resources, time, and training to correctly code information received from secondary care.

## Discussion

### Summary

This QI project provides a detailed assessment of cancer-related clinical coding in NCL primary care across a population of 1.4 million adults. Our findings show that although some demographic data such as ethnicity and language are well captured (coding completeness), many other important codes across the cancer pathway—especially those relating to social determinants of health, cancer treatments, and postdiagnosis care—remain inconsistently coded or significantly absent from primary care records.

Coding quality was strongly influenced by the presence of national or local incentives (such as QOF), which drove completeness for certain indicators like ethnicity, cancer diagnosis, and CCRs. In contrast, areas not linked to performance payments or formalized data capture processes, such as cancer treatment, staging, and screening follow-up, showed substantial gaps. These differences signal that current coding behavior in primary care is shaped by system design, contractual levers, and administrative capacity.

Further qualitative insights helped contextualize these patterns, showing that GPs often prioritize coding activities relevant to their daily clinical workflow or incentivized tasks. Key barriers to good coding included complex and inconsistent coding systems, limited opportunities to update data, and limited structured information sharing from secondary care.

Overall, the findings demonstrate that improving cancer coding quality in primary care requires more than local process changes; it will require a coordinated national approach that includes clearer coding standards, automation, and alignment of incentives.

### Interpretation

Our findings align with existing literature relating to cancer data coding in primary care. For example, our observation that ethnicity coding was high at 86.5% (912,679/1,055,083), is consistent with other studies using patient electronic records. This is similar to 78.2% ethnicity coding reported in NHS primary care records in 2022 [40]. These high levels may be related to incentive schemes (QOF) to improve completeness in England [41]. Similarly, 68.7% (898,023/1,307,601) completeness for language coding aligns with findings that emphasize the need for comprehensive demographic information to support better patient health outcomes [42].

The minimal recording of employment status (29,848/1,229,644, 2.4%) and the moderate completeness of BMI data (485,660/1,159,241, 41.9%) underscore challenges in capturing socioeconomic and health metrics. These gaps are consistent with literature indicating that certain health data, such as employment status, are often underreported in primary care settings [43]. Our findings on smoking status (530,720/1,185,812, 44.8%) and alcohol consumption (222,753/1,236,580, 18%) coding completeness, which show significant borough variation, also reflect widespread underrecording of these factors in patient records [44]. This may reflect discomfort in approaching conversations about lifestyle factors as well as lack of time or resources, particularly in more deprived areas [12,45].

The variation in cancer screening data across pathways, particularly the fragmented coding for breast screening, highlights the complexities in capturing screening information. Rafi et al [46] also found that a wide range of codes were used to document a family history of breast cancer in primary care, leading to inconsistencies in data quality and the potential for misclassification of risk. This finding may relate to primary care’s role and responsibilities in screening, where their involvement varies significantly across different cancer types [47]. While primary care plays a structured role in bowel and cervical screening (underpinned by funding and agreed processes nationally), breast cancer screening activity is not delivered or incentivized in primary care.

New cancer diagnoses were also undercoded, at 87% (4604/5260) completeness. This aligns with findings from the Netherlands, where only 60.6% of cancer cases were coded according to the national registry [9]. Undercoding is linked to reliance on unstructured secondary care letters and a lack of coding incentives [8,9]. Cancer treatment and staging data were also undercoded, and there was not any existing evidence to compare against.

The recording of CCRs (3953/5982, 66% of eligible patients) closely mirrors the proportion of coded diagnoses, suggesting a correlation between diagnosis coding and care review documentation. However, concerns exist that high completeness rates reflect a “tick-box” approach driven by financial incentives for coding “cancer care review” [48]. Documentation of broader personalized cancer care indicators, such as care plans and holistic needs assessments, was minimal (1%-2%), highlighting gaps in comprehensive cancer care documentation. This aligns with studies calling for broader, patient-centered metrics to sustain confidence in cancer registries [49]. National CCR templates embedded in GP electronic records [50] support additional quality indicator completion alongside CCRs, promoting standardization [51]. The lack of broader care indicators being coded suggests that these templates are not embedded in daily practice.

While this project was conducted in NCL, the findings have broader relevance across other similar primary care settings. Although population demographics vary between regions, the core structures, processes, and incentives that shape GP coding behaviors are largely consistent across the NHS and similar international health care systems. Primary care operates within a standardized framework of national policies, contractual obligations, and clinical systems, meaning that the challenges and opportunities identified in this study are likely to be mirrored elsewhere. As such, our findings provide valuable insights for informing coding improvement initiatives beyond our study region, with potential applicability across primary care systems nationally.

### Limitations

Two boroughs were excluded due to nonengagement; however, the dataset remained robust with high completeness in other areas, minimizing bias. The findings should be interpreted cautiously for nonsubmitting practices. Additionally, our quantitative data searches were completed by GP Federation’s IT team, but their searches could not fully align with our specifications. This left gaps in key cancer care metrics, such as patients with cancer on stratified follow-up pathways or active surveillance; alternative data sources such HealtheIntent (the ICBs’ provider for linked datasets across the NHS in NCL until September 2025), helped fill in some gaps.

### Conclusions

The QI project has provided a unique and detailed insight into the many dimensions of cancer coding across the whole pathway in primary care and sheds light on many factors that underpin variation and coding preference.

We have developed recommendations based on our findings aimed at primary care providers, commissioners, ICBs’ digital teams [52], cancer screening teams and the National Cancer team [53].

### Implications for Practice

Textbox 1 outlines practical recommendations to enhance primary care data management, integration with secondary care, and overall service quality. The focus is on standardizing coding practices, improving information flow, and leveraging data for informed decision-making and patient care improvements.

**Implications for practice.**

**Strengthen data infrastructure in primary care**

- Develop a structured data framework using SNOMED CT (Systematized Nomenclature of Medicine – Clinical Terms) with a minimum dataset and distinct code sets, aligning with secondary care processes. This will enable better use of primary care data for analysis, epidemiology, and health care improvement.

**Enhance breast screening integration with primary care**

- Transition to electronic breast screening outcome reports for general practitioners (GPs), standardize national breast screening activity codes within SNOMED CT, and define and resource primary care’s role in patient follow-up and nonattendance intervention. These changes will improve the accuracy and efficiency of breast screening data and ensure clear responsibility for patient engagement.

**Improve secondary care information for primary care**

- Standardize hospital discharge letters to clearly indicate new diagnoses, multidisciplinary team outcomes, and care plans, ensure clinical coding aligns with SNOMED CT; prioritize consistent implementation of end-of-treatment summaries; and link secondary care data directly to primary care [27-29]. This recommendation aligns with the national strategy [54,55]. This will reduce duplication, enhance continuity of care, and improve data quality for patient management.

**Standardize primary care coding practices**

- Employ coding professionals in primary care, align screening activity codes, update bowel screening codes to reflect the transition from fecal occult blood to fecal immunochemical tests, and educate the workforce on the value of high-quality coding. These steps will improve consistency, accuracy, and the usefulness of coded data for patient care and service planning.

**Optimize patient registration processes**

- Automate the integration of coded demographic, behavioral, and risk factor data from National Health Service Digital’s updated registration forms (Patient Registration Form 1) [56] into primary care systems and allocate resources for implementation. This will ensure more complete and accurate patient information from the outset.

**Improve Quality Outcomes Framework rules and transparency**

- Make Quality Outcomes Framework rule changes trackable over time and provide clearer navigation and updates [57,58]. This will help those involved in service improvement and research to understand and respond to coding changes more effectively.

**Implement primary care coding audits**

- Introduce National Health Service England–funded coding audits, assess data quality and completeness, flag nonrecommended codes, and cross-reference with national datasets. This will improve coding accuracy, highlight inconsistencies, and enhance data reliability.

**Develop an analytics dashboard**

- Create a live dashboard to track trends, profile data, and support quality improvement, leveraging the London Health Data Service (launched in June 2025) [59]. This will provide real-time insights into primary care data, supporting better decision-making and service planning.

**Facilitate knowledge sharing**

- Identify regions with superior data completeness and share successful quality improvement initiatives across London. This will promote best practices and drive improvements in data quality and patient care across the system.

### Funding

This study did not receive external funding from any public, commercial, or not-for-profit sources. The Enfield Federation was commissioned to build the data searches, run these across NCL GP practices, and extract the data. SB’s time was funded by the NCL Cancer Alliance to support the qualitative analysis. GB acknowledges funding from Barts Charity. No funding body was involved in study design, data collection, analysis, interpretation, or manuscript preparation.

## Appendix

Multimedia Appendix 1 Supplementary (clean) 30.10.25.

## Abbreviations

CCR: cancer care review
COREQ: Consolidated Criteria for Reporting Qualitative Research
EMIS: Egton Medical Information Systems
FIT: fecal immunochemical test
FOB: fecal occult blood
gFOBt: guaiac fecal occult blood testing
GP: general practitioner
ICB: integrated care board
ICD-10: International Statistical Classification of Diseases, Tenth Revision
NCL: North Central London
NCRAS: National Cancer Registration and Analysis Service
NHS: National Health Service
PCN: primary care network
QI: quality improvement
QOF: Quality Outcomes Framework
SNOMED CT: Systematized Nomenclature of Medicine – Clinical Terms

## Data Availability

The datasets generated and analyzed during this study are available from the corresponding author on reasonable request.
