# Supplementary material for: Quality of Cancer-Related Clinical Coding in Primary Care in North Central London: Mixed Methods Quality Improvement Project
Source: J Med Internet Res. 2026 Jan 7;28:e73205. doi: 10.2196/73205 (PMC12824575; doi:10.2196/73205)
Supplement: Multimedia Appendix 1 [file jmir_v28i1e73205_app1.docx]

SUPPLEMENTARY FILE

##### *Table 1:* Revised Standards for QUality Improvement Reporting Excellence (SQUIRE 2.0) checklist - completed with page numbers

|  |  | |  |
| --- | --- | --- | --- |
| **Text section and item name** | | **Page/line no(s).** | |
|  | | **info is located** | |
| **Title and abstract** | |  | |
| 1. **Title** | | 1 | |
| Indicate that the manuscript concerns an initiative to improve healthcare (broadly defined to include the quality, safety, effectiveness, patient-centredness, timeliness, cost, efficiency and equity of healthcare). | |  | |
|  | |  | |
| 2. **Abstract** | | 1-2 | |
| a. Provide adequate information to aid in searching and indexing. | |  | |
| b. Summarise all key information from various sections of the text using the abstract format of the intended publication or a structured summary such as: background, local problem, methods, interventions, results, conclusions. | |  | |
|  | |  | |
| **Introduction: Why did you start?** | |  | |
| 3. **Problem description** - Nature and significance of the local problem. | | 2 | |
| 4. **Available knowledge** - Summary of what is currently known about the problem, including relevant previous studies. | | 3 | |
| 5. **Rationale** - Informal or formal frameworks, models, concepts and/or theories used to explain the problem, any reasons or assumptions that were used to develop the intervention(s) and reasons why the intervention(s) was expected to work | | 3-4 | |
| 6. **Specific aims** - Purpose of the project and of this report. | | 4 | |
|  | |  | |
| **Methods: What did you do?** | |  | |
| 7. **Context** - Contextual elements considered important at the outset of introducing the intervention(s). | | 4 | |

| 8. **Intervention(s)** | 5 |
| --- | --- |
| a. Description of the intervention(s) in sufficient detail that others could reproduce it. |  |
| b. Specifics of the team involved in the work. |  |
| 9. **Study of the intervention(s)** | 5 |
| a. Approach chosen for assessing the impact of the intervention(s). |  |
| b. Approach used to establish whether the observed outcomes were due to the intervention(s). |  |
| 10. **Measures** | 6-7 |
| a. Measures chosen for studying processes and outcomes of the intervention(s), including rationale for choosing them, their operational definitions and their validity and reliability. |  |
| b. Description of the approach to the ongoing assessment of contextual elements that contributed to the success, failure, efficiency and cost. |  |
| c. Methods employed for assessing completeness and accuracy of data. |  |
| 11. **Analysis** | 7-14 |
| a. Qualitative and quantitative methods used to draw inferences from the data. |  |
| b. Methods for understanding variation within the data, including the effects of time as a variable. |  |
| 12. **Ethical considerations** - Ethical aspects of implementing and studying the intervention(s) and how they were addressed, including, but not limited to, formal ethics review and potential conflict(s) of interest. | 4 |
|  |  |
| **Results: What did you find?** |  |
| 13. **Results** | 13 - 26 |
| a. Initial steps of the intervention(s) and their evolution over time (eg, time-line diagram, flow chart or table), including modifications made to the intervention during the project. |  |
| b. Details of the process measures and outcomes. |  |
| c. Contextual elements that interacted with the intervention(s). |  |
| d. Observed associations between outcomes, interventions and relevant contextual elements. |  |
| e. Unintended consequences such as unexpected benefits, problems, failures or costs associated with the intervention(s). |  |
| f. Details about missing data. | 11 |
|  |  |
| **Discussion: What does it mean?** | **26** |
| 14. **Summary** | 26 |
| a. Key findings, including relevance to the rationale and specific aims. |  |
| b. Particular strengths of the project. |  |
|  |  |
| 15. **Interpretation** | 27 |
| a. Nature of the association between the intervention(s) and the outcomes. |  |
| b. Comparison of results with findings from other publications. |  |
| c. Impact of the project on people and systems. |  |
| d. Reasons for any differences between observed and anticipated outcomes, including the influence of context. |  |
| e. Costs and strategic trade-offs, including opportunity costs. |  |
|  |  |
| 16. **Limitations** | 28 |
| a. Limits to the generalisability of the work. |  |
| b. Factors that might have limited internal validity such as confounding, bias or imprecision in the design, methods, measurement or analysis. |  |
| c. Efforts made to minimise and adjust for limitations. |  |
|  |  |
| **Conclusions** | 28 |
| a. Usefulness of the work. |  |
| b. Sustainability. |  |
| c. Potential for spread to other contexts. |  |
| d. Implications for practice and for further study in the field. | 28 |
| e. Suggested next steps. |  |
|  |  |
| **Other information** |  |
| 18. **Funding** - Sources of funding that supported this work. Role, if any, of the funding organisation in the design, implementation, interpretation and reporting. | 30 |
|  |  |

*Table 2.* Barriers to clinical coding in primary care

|  | BARRIER | EXPLANATION |
| --- | --- | --- |
| 1 | Lack of standardised coding practices | No existing standard for documenting cancer activity |
| 2 | Lack of staff to carry out coding | The person undertaking the coding varies significantly across GP practices (e.g., GPs, practice nurses, administrative clerks, practice manager etc) and hence their training/experience. |
| 3 | Limited knowledge and training in medical coding | Clinical coding is not part of the GP curriculum and in a GP trainee’s training journey, they may not be taught the importance of coding accuracy and its implications for service planning and quality improvement. Clinical coding it not held in one part of the system (i.e. there is no official coder across primary care) - it is everyone's job to know and apply good clinical coding. New healthcare professionals in primary care may not be inducted/taught re clinical coding practices. |
| 4 | Insufficient time and resourcing towards coding | Prioritisation of clinical coding varies within and across practices. |
| 5 | Difficulty capturing all relevant information to code | The data transfer from paper/digital platforms (I.e., non-primary care providers) to the electronic health care record is not systematic. Screening letters or Trusts letters do not send standard letters and diagnosis codes are not always included, therefore primary care staff need to extract the relevant clinical information and assign a code. |
| 6 | Complexity of coding | Diversity in coding options for a singular event means more than one code can be used (e.g. SNOMED has various options for coding one type of cancer). |
| 7 | Code doesn't exist | SNOMED code might not exist for that type of activity. You can author SNOMED codes (which is a specialist skill and very few people have it) or request new codes to be created |
| 8 | Insufficient software support | Tools exist to support coding and uptake is variable |
| 8 | Lack of incentive/benefit | No financial incentives to capture a diverse set of data fields in primary care – in contrast secondary care are required to capture diagnostic and procedural codes to generate payment |

*Figure 1*: PROJECT TIMELINE overview


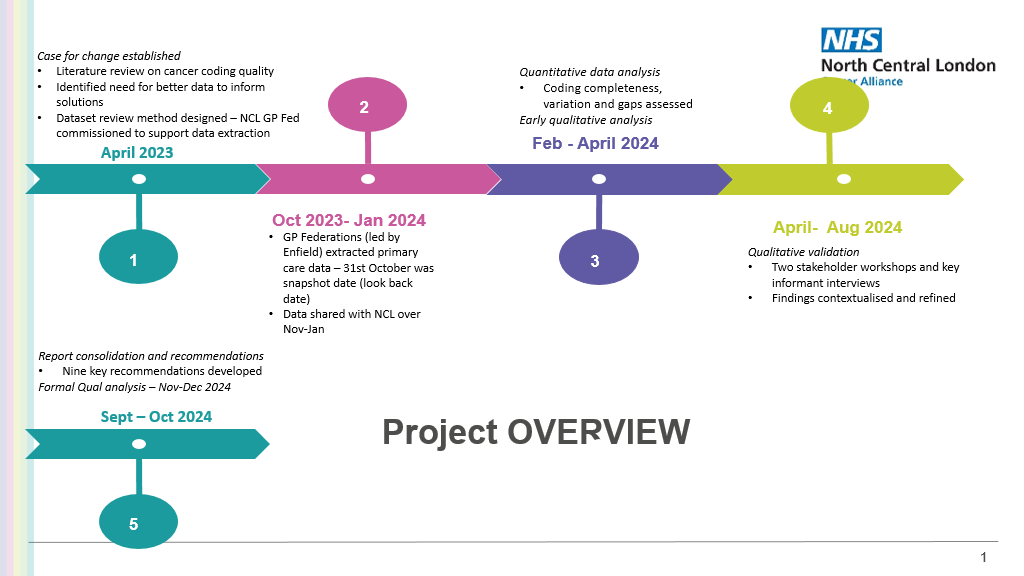


Table 3. Consolidated criteria for reporting qualitative research

Tong A, Sainsbury P, Craig J. Consolidated criteria for reporting qualitative research (COREQ): a 32-item checklist for interviews and focus groups. International Journal for Quality in Health Care. 2007. Volume 19, Number 6: pp. 349 – 357.

| Topic | Item | Guide Questions/Description | Reported in Section (Page No.) |
| --- | --- | --- | --- |
| Domain 1: Research team and reflexivity | | | |
| Personal characteristics | | | |
| Interviewer/facilitator | 1 | Which author/s conducted the interview or focus group? | Qualitative data collection (page 6) |
| Credentials | 2 | What were the researcher’s credentials? E.g. PhD, MD | Qualitative data collection (page 6) |
| Occupation | 3 | What was their occupation at the time of the study? | Qualitative data collection  (page 6) |
| Gender | 4 | Was the researcher male or female? | Qualitative data collection (page 6) |
| Experience and training | 5 | What experience or training did the researcher have? | Qualitative data collection  (page 6) |
| Relationship with participants | | |  |
| Relationship established | 6 | Was a relationship established prior to study commencement? | Design  (page 4) |
| Participant knowledge of the interviewer | 7 | What did the participants know about the researcher? e.g. personal goals, reasons for doing the research | Appendix: Reflexivity statement |
| Interviewer characteristics | 8 | What characteristics were reported about the inter viewer/facilitator? e.g. Bias, assumptions, reasons and interests in the research topic | Appendix: Reflexivity statement |
| Domain 2: Study design | | |  |
| Theoretical framework | | |  |
| Methodological orientation and Theory | 9 | What methodological orientation was stated to underpin the study? e.g. grounded theory, discourse analysis, ethnography, phenomenology, content analysis | Appendix: Reflexivity statement |
| Participant selection | | |  |
| Sampling | 10 | How were participants selected? e.g. purposive, convenience, consecutive, snowball | Design  (page 4) |
| Method of approach | 11 | How were participants approached? e.g. face-to-face, telephone, mail, email | Appendix: Reflexivity statement |
| Sample size | 12 | How many participants were in the study? | Design (page 4) |
| Non-participation | 13 | How many people refused to participate or dropped out? Reasons? | n/a |
| Setting | | |  |
| Setting of data collection | 14 | Where was the data collected? e.g. home, clinic, workplace | Qualitative data collection (page 6) |
| Presence of non-participants | 15 | Was anyone else present besides the participants and researchers? | Appendix: Reflexivity statement |
| Description of sample | 16 | What are the important characteristics of the sample? e.g. demographic data, date | Results - Qualitative Stakeholder workshops (page 8-9) |
| Data collection | | |  |
| Interview guide | 17 | Were questions, prompts, guides provided by the authors? Was it pilot tested? | Qualitative data collection (page 6) |
| Repeat interviews | 18 | Were repeat inter views carried out? If yes, how many? | n/a |
| Audio/visual recording | 19 | Did the research use audio or visual recording to collect the data? | Qualitative data collection (page 6) |
| Field notes | 20 | Were field notes made during and/or after the interview or focus group? | Qualitative data analysis (page 7) |
| Duration | 21 | What was the duration of the interviews or focus group? | Results - Qualitative Stakeholder workshops (page 8-9) |
| Data saturation | 22 | Was data saturation discussed? | n/a |
| Transcripts returned | 23 | Were transcripts returned to participants for comment and/or correction? | Qualitative data collection (page 6) |
| Domain 3: analysis and findings | | | |
| Data analysis | | | |
| Number of data coders | 24 | How many data coders coded the data? | Qualitative data analysis (page 7) |
| Description of the coding tree | 25 | Did authors provide a description of the coding tree? | Qualitative data analysis (page 7) |
| Derivation of themes | 26 | Were themes identified in advance or derived from the data? | Qualitative data analysis (page 7) |
| Software | 27 | What software, if applicable, was used to manage the data? | Qualitative data analysis (page 7) |
| Participant checking | 28 | Did participants provide feedback on the findings? | Qualitative data collection (page 6) |
| Reporting | | | |
| Quotations presented | 29 | Were participant quotations presented to illustrate the themes/findings? Was each quotation identified? e.g. participant number | Qualitative results  (pages 14-15, 19-20, 24) |
| Data and findings consistent | 30 | Was there consistency between the data presented and the findings? | Qualitative results (pages 14-15, 19-20, 24) |
| Clarity of major themes | 31 | Were major themes clearly presented in the findings? | Qualitative results (page 14-15, 19-20, 24) |
| Clarity of minor themes | 32 | Is there a description of diverse cases or discussion of minor themes? | Qualitative results (pages 14-15, 19-20, 24) |

*Table 4*. Qualitative data sources (interviews and workshops - which were held remotely via MS Teams) including times, duration and attendance

| **Session** | **Date** | **Duration (mins)** | **Attendees** |
| --- | --- | --- | --- |
| GP Federation group validation interview meeting 1 | 29/04/2024 | 72:27 | AB (Interviewer), GR (Research team facilitator)  2 x federation representatives with digital and operational expertise |
| GP Federation group validation interview meeting 2 | 11/06/2024 | 54:82 | AB (Interviewer), GR (Research team facilitator)  3 x federation representatives with digital, clinical and operational expertise |
| Stakeholder workshop 1 - Held as one group for key information and split into two smaller groups for theme discussion before coming back together as one group at the end to discuss key reflections | 02/07/2024 | 113:51 | AB (Interviewer) KT (Research team facilitator) GR (Research team facilitator)  Room 1  4 GPs  2 Academic Researchers  2 Project Managers*  1 Programme Lead  Room 2  3 GPs  2 Programme Lead  1 Academic Researcher |
| Stakeholder workshop 2 - Held as one group throughout the session | 01/08/2024 | 114:73 | AB (Interviewer) KT (Research team facilitator) GR (Research team facilitator)  6 GPs  1 Academic Research  1 IT Team member  1 Programme Lead  1 Project Manager  1 Head of Cancer Commissioning |

#####

##### *Project manager = responsible for a specific programme of work in the alliance, which sits beneath a cancer programme. E.g., improve bowel screening in specific populations.

##### **Programme Manager = Responsible to deliver against a whole programme of work in the Cancer Alliance i.e. Prevention and screening or Personalised Cancer Care.

*Box 1*: Extracted search terms in their three formats for different users

| 1: EMIS Web searches with underpinning terms and parent codes (PDF)  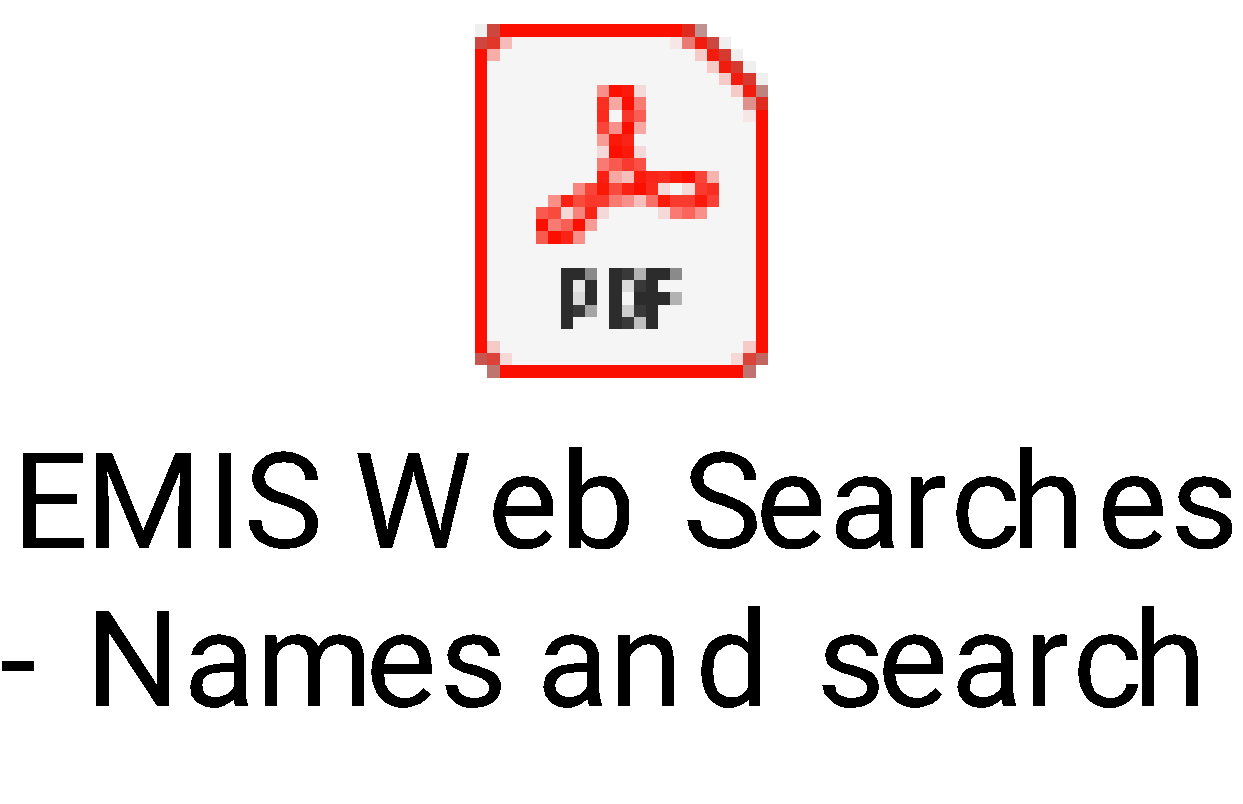  2: EMIS Web xml file - to run searches in EMIS Web systems  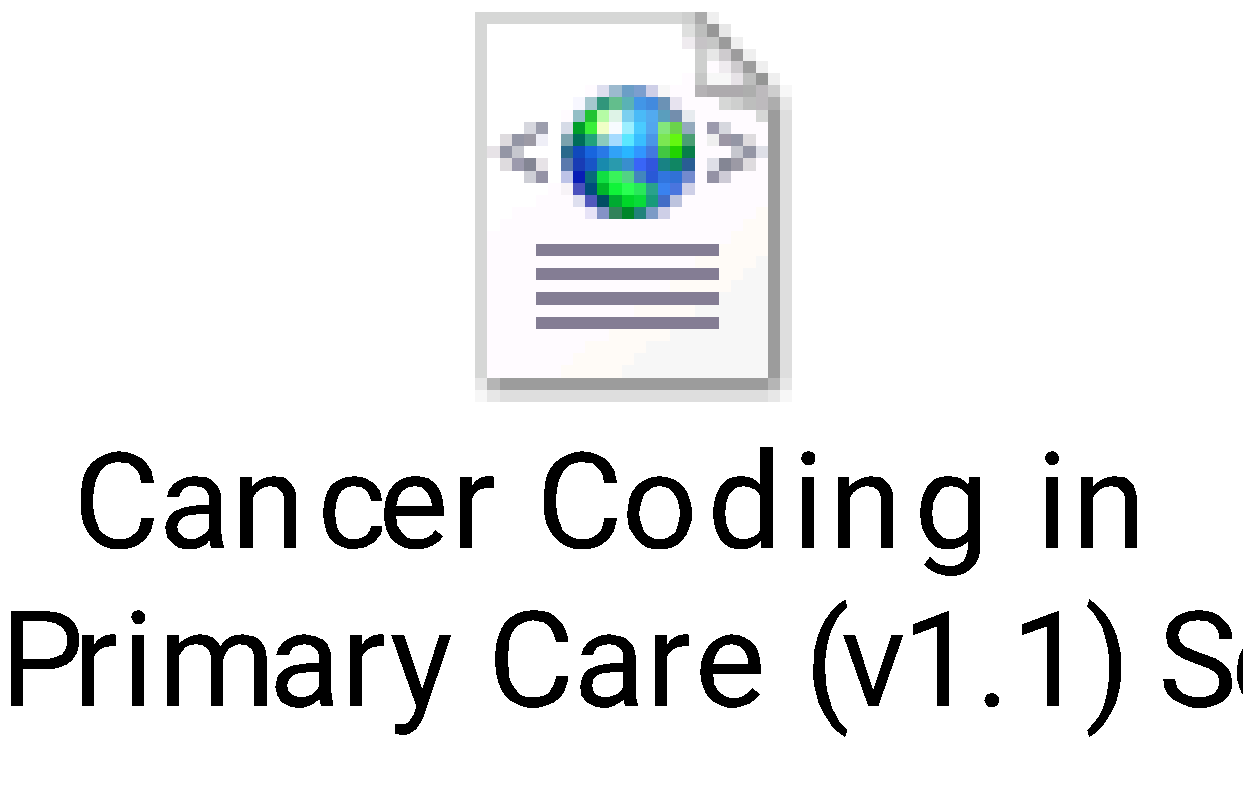  3. Deconstructed XML code  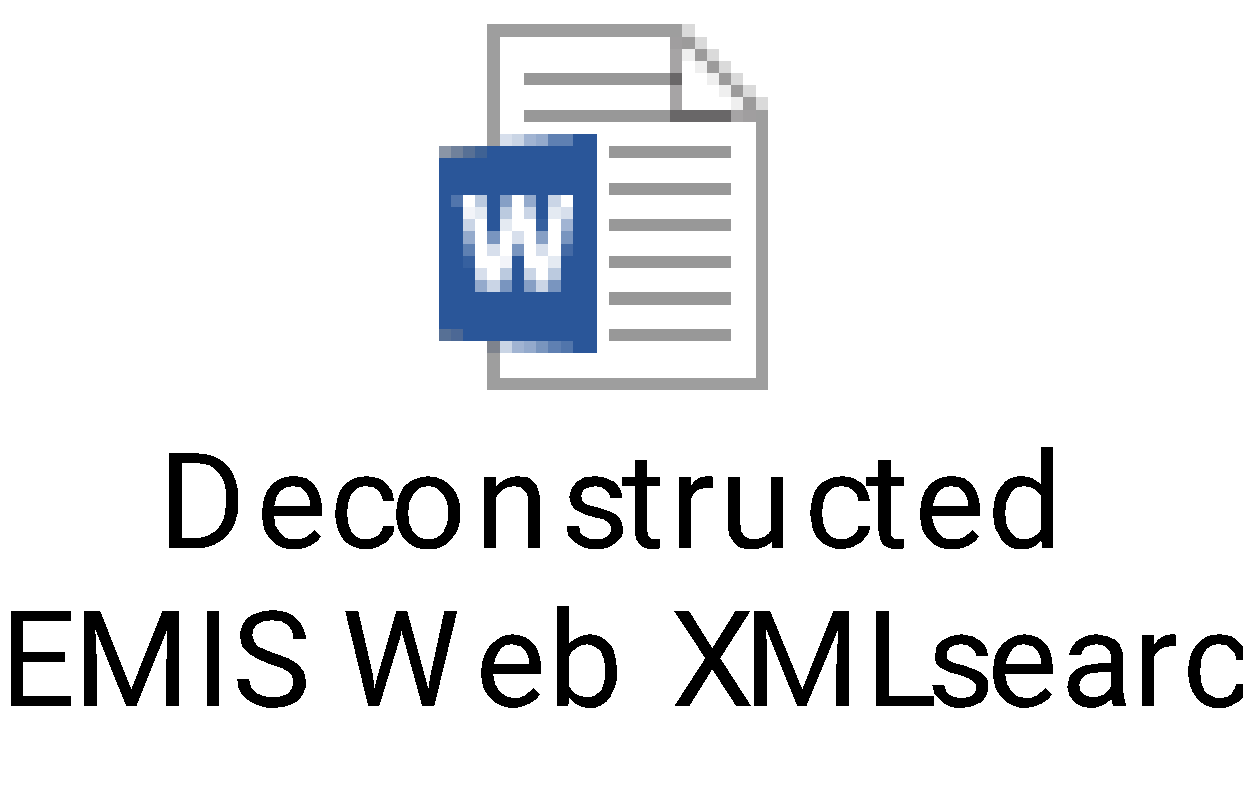 |
| --- |

*Table 5.* Baseline adult populations across NCL by borough (April 2024)

|  | Barnet | Camden | Enfield | Haringey | Islington | **NCL** |
| --- | --- | --- | --- | --- | --- | --- |
| Population 18 and over | 352,882 | 304,211 | 281,807 | 282,194 | 186,871 | **1,407,965** |

*Table 6.* Data report returns table by the GP federations

| Report reference | Report Name | Barnet | Camden | Camden Health Evolution | Enfield | Haringey | Islington | Islington North 2 |
| --- | --- | --- | --- | --- | --- | --- | --- | --- |
| 1 | Ethnic Origin (Coding) |  | Y | Y | Y | Y | Y |  |
| 1a | Ethnic Origin (Patient details) |  | Y | Y | Y | Y | Y |  |
| 2 | Fast Track Referral Coding | Y | Y | Y | Y | Y | Y |  |
| 3 | Malignant Neoplastic Disease | Y | Y | Y | Y | Y | Y |  |
| 3a | Treatment regime (if coded) | Y | Y | Y | Y | Y | Y |  |
| 3a2 | Cancer Care Review | Y | Y | Y | Y | Y | Y |  |
| 3b | Malignancy Stage Coding | Y | Y | Y | Y | Y | No data |  |
| 4a | Alcohol consumption record | Y | Y | Y | Y | Y | Y |  |
| 4b | Current smoker (recorded in last 24m) | Y | Y | Y | Y | Y | Y |  |
| 4c | Electronic cigarette user | Y | Y | Y | Y | Y | Y |  |
| 4d | Any smoking status (recorded in last 24m) | Y | Y | Y | Y | Y | Y |  |
| 4e | Family history of neoplasm | Y | Y | Y | Y | Y | Y |  |
| 4f | Main language spoken | Y | Y | Y | Y | Y | Y |  |
| 4g | Weight and-or BMI recorded | Y | Y | Y | Y | Y | Y |  |
| 4h | Employment status | Y | Y | Y | Y | Y | Y |  |
| 4i | Environmental pollutants | Y | Y | Y | Y | Y | Y |  |
| 4j | Has a carer | Y | Y | Y | Y | Y | Y |  |
| 5a | Cancer-Bowel - 60-74yrs did not return screening kit | Y | Y | Y | Y | Y | Y |  |
| 5b | Cancer-Bowel - 60-74yrs screened | Y | Y | Y | Y | Y | Y |  |
| 5b1 | Cancer-Bowel abnormal result | Y | Y | Y | Y | Y | Y |  |
| 6a | Cancer-Breast - 50-70yrs screened | Y | Y | Y | Y | Y | Y |  |
| 6a1 | Cancer-Breast - 50-70yrs abnormal result | Y | Y | Y | Y | No data | Y |  |
| 6a2 | Cancer-Breast - 50-70yrs normal result | Y | Y | Y | Y | Y | Y |  |
| 6a3 | Cancer-Breast cancer detected | Y | Y | Y | Y | Y | Y |  |
| 7a1 | Cancer-Cervical - 25-49y adequate smear | Y | Y | Y | Y | Y | Y |  |
| 7b1 | Cancer-Cervical - 50-64y adequate smear | Y | Y | Y | Y | Y | Y |  |

RED: Search not run

No data: search was run, no data generated

**Explanation for failed searches run by the federation**

The technical team would liaise with EMIS Web help desk to try and resolve these. Unfortunately, it was not clear why the failure occurred and hence solutions were not found. Several explanations were offered by EMIS Web such as the cancer searches being less of a priority beneath other contractual searches, that the search had a limited time to run and if they superseded it then failure was the result, or the search scheduler itself became stalled. EMIS Web advised to run the searches practice by practice which was not feasible and defied the point of at scale enterprise searches. In the end, the searches needed to be run multiple times until they finally succeeded in generating an extract. The sites/federations that persisted or had inhouse IT teams to manage running the 26 report searches succeeded in obtaining more complete data than those that did not.

*Table 7.* Total number and names of the reports (searches) that were run in each borough in North Central London, and the proportion of practices that submitted their data e.g. 100% = all practices in that borough sent through data on X report.

| Report reference | Report Name | Barnet  (48) | Camden  (32) | Enfield  (30) | Haringey  (34) | Islington  (23) |
| --- | --- | --- | --- | --- | --- | --- |
| 1 | Ethnic Origin (Coding)* | Not submitted | 100% | 100% | 100% | 100% |
| 1a | Ethnic Origin (Patient details)* | Not submitted | 100% | 90% | 100% | 100% |
| 2 | Fast Track Referral Coding | 48% | 100% | 100% | 100% | 100% |
| 3 | Malignant Neoplastic Disease | 48% | 100% | 100% | 100% | 100% |
| 3a | Treatment regime (if coded) | 35% | 66% | 77% | 53% | 65% |
| 3a2 | Cancer Care Review | 48% | 100% | 100% | 100% | 100% |
| 3b | Malignancy Stage Coding | 2% | 6% | 7% | 3% | Not submitted |
| 4a | Alcohol consumption record | 48% | 100% | 100% | 100% | 100% |
| 4b | Current smoker (recorded in last 24m) | 48% | 100% | 100% | 100% | 100% |
| 4c | Electronic cigarette user | 48% | 100% | 100% | 100% | 100% |
| 4d | Any smoking status (recorded in last 24m) | 48% | 100% | 77% | 100% | 100% |
| 4e | Family history of neoplasm | 48% | 100% | 100% | 100% | 100% |
| 4f | Main language spoken | 96% | 100% | 63% | 100% | 100% |
| 4g | Weight and-or BMI recorded | 46% | 100% | 73% | 100% | 100% |
| 4h | Employment status | 48% | 100% | 100% | 100% | 100% |
| 4i | Environmental pollutants | 42% | 91% | 73% | 79% | 87% |
| 4j | Has a carer | 48% | 100% | 100% | 100% | 100% |
| 5a | Cancer-Bowel - 60-74yrs did not return screening kit | 100% | 100% | 100% | 100% | 100% |
| 5b | Cancer-Bowel - 60-74yrs screened | 100% | 100% | 87% | 100% | 100% |
| 5b1 | Cancer-Bowel abnormal result | 100% | 100% | 97% | 100% | 100% |
| 6a | Cancer-Breast - 50-70yrs screened | 100% | 100% | 100% | 100% | 100% |
| 6a1 | Cancer-Breast - 50-70yrs abnormal result | 92% | 91% | 73% | Not submitted | 87% |
| 6a2 | Cancer-Breast - 50-70yrs normal result | 100% | 100% | 97% | 100% | 100% |
| 6a3 | Cancer-Breast cancer detected | 96% | 91% | 87% | 88% | 87% |
| 7a1 | Cancer-Cervical - 25-49y adequate smear | 100% | 100% | 80% | 100% | 100% |
| 7b1 | Cancer-Cervical - 50-64y adequate smear | 100% | 100% | 100% | 100% | 100% |

**Ethnic origin was shown twice as within EMIS Web users can record this data in 2 different sections. One is via ‘patient details’, this is stored in the registration information, or the other option is via clinical coding (SNOMED). It is uncertain why there are 2 options with an assumption it could be a historical function of EMIS Web.*

*Box 2*. Reflexivity statement

Stakeholders were invited through advertising the remote workshops through the NCL GP bulletin, NCL GP website, NCL Practice managers networks, direct emails and NCL GP WhatsApp groups. The advert described how the aim of the project was to improve the quality and consistency of clinical coding. Other members of the research team were present during the workshops to facilitate discussion or provide explanations of the data presented.
The researchers who conducted the qualitative analysis (SB, GBB, both female) have previous experience conducting qualitative research. SB is a behavioural science PhD student and GBB is a Reader in Applied Health Research. Both researchers do not have clinical expertise. This facilitated an objective approach towards understanding barriers to clinical coding. The data were analysed through a critical realist perspective, assuming that the observed completeness and consistency of clinical coding in quantitative data can be understood by exploring the subjective realities of individuals involved in the clinical workflow of patient data capture and coding.(Pilgrim, 2019) SB listened to all video-recordings and checked the transcripts for accuracy to become familiar with the study context and dataset, before coding the data. Data was coded if it specifically related to factors influencing the quality of cancer clinical coding in primary care.


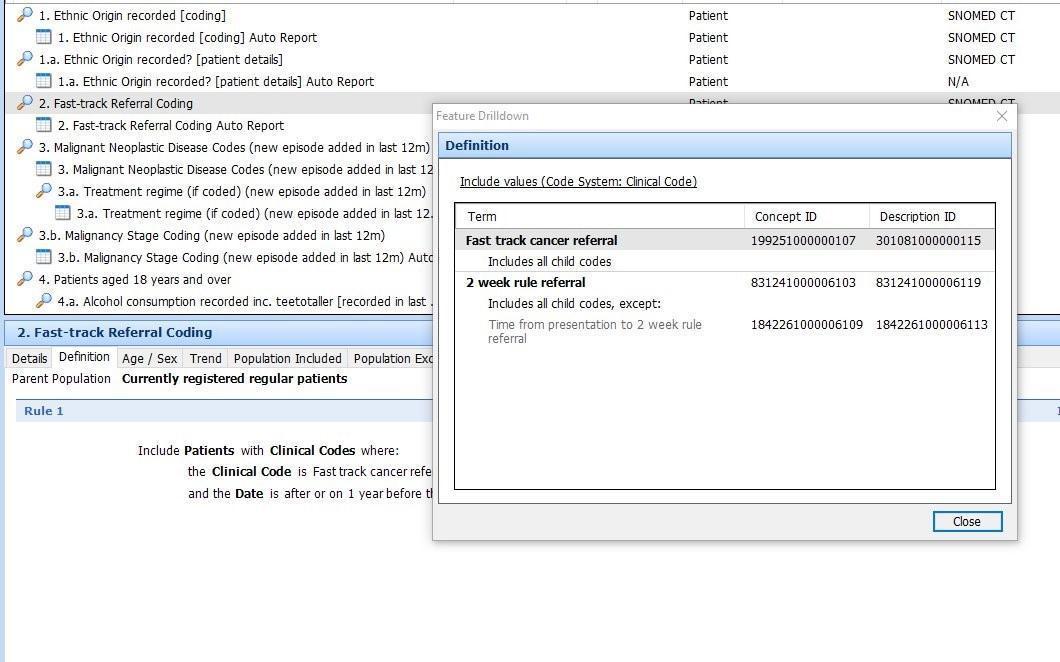


*Figure 2* EMIS Web searches – fast track cancer referrals against the backdrop of various searches defined

*Table 8.* The themes by which combined data (quantitative and qualitative) findings is presented and shown according to data source, pathway stage and data domain

| No | DATA SOURCE | PATHWAY STAGE AREA | DATA DOMAIN |
| --- | --- | --- | --- |
| Theme 1: PRE-CANCER | | | |
| 1a | Data capture at GP registration | Demographics, Physical characteristics and Cancer risk factors | Ethnicity, Language spoken, Employment status, Body weight, BMI, Smoking status, Alcohol consumption, Family history of cancer, Environmental exposures linked to cancer risk (Asbestos, Arsenic, UV light man made, Sun exposure) |
| 1b | Data capture in the clinical records during any primary care interaction with patient | Demographics, Physical characteristics, Cancer risk factors and suspected cancer referrals | Ethnicity, Language spoken, Employment status, Body weight, BMI, Smoking status, Alcohol consumption, Family history of cancer, Environmental exposures linked to cancer risk (Asbestos, Arsenic, UV light man made, Sun exposure), Suspected cancer referrals |
| Theme 2: SCREENING | | | |
| 2 | Mixed picture | Screening | Cervical cancer screening, Bowel cancer screening, Breast cancer screening |
| Theme 3: POST-CANCER DIAGNOSIS | | | |
| 3a | Data generated in secondary care | New cancer diagnosis, cancer stage and location, cancer treatment, cancer treatment monitoring | Chemotherapy, hormone therapy, radiotherapy, immunotherapy, active monitoring, MDT discussion, genomic testing. |
| 3b | Data capture in the clinical records during any primary care interaction with patient – post diagnosis | Personalised cancer care, cancer surveillance and follow up | Cancer care review, cancer care plan discussed, carer, Next of kin, HNA, Active monitoring, Stratified follow up |

*Table 9.* Theme 1**:** Completeness, code diversity and validation, and granularity of coding.

| **REPORT NAME** | **Time Period** | **Completeness**  **and**  **Code diversity** | | **Code validation** | **Granularity -** ​(Note: granularity - top 5 codes - if 5 were not available then min number used were reviewed) | | | | |
| --- | --- | --- | --- | --- | --- | --- | --- | --- | --- |
|  |  | Completeness - % of eligible patients with code captured | No of unique SNOMED/ EMIS codes in search | EMIS code capture coverage as a proportion of comparator | Code prevalence - TOP 5 | % or Total no. of all codes captured in NCL | SNOMED Parent | SNOMED child | Non-SNOMED |
| Ethnic Origin | Recorded ever | 86.5% | 375 | 95.4% | Other White background - ethnic category 2001 census | 18.6% |  | x |  |
|  |  |  |  |  | British or mixed British - ethnic category 2001 census | 13.7% |  |  | x |
|  |  |  |  |  | White British - ethnic category 2001 census | 9.0% |  | x |  |
|  |  |  |  |  | African - ethnic category 2001 census | 5.8% |  | x |  |
|  |  |  |  |  | Chinese - ethnic category 2001 census | 3.7% |  | x |  |
| Main language spoken | Recorded ever | 68.7% | 338 | 109.4% | Main spoken language English | 58.7% |  | x |  |
|  |  |  |  |  | Main spoken language NOS | 5.4% |  |  | x |
|  |  |  |  |  | Main spoken language Turkish | 3.1% |  | x |  |
|  |  |  |  |  | Main spoken language Spanish | 2.4% |  | x |  |
|  |  |  |  |  | Main spoken language Polish | 1.8% |  | x |  |
| Employment status | Oct 2021 – Oct 2023 | 2.4% | 87 | No comparator for coding prevalence of this data item sourced | Unemployed | 39.5% | x |  |  |
|  |  |  |  |  | Stress at work | 17.0% | x |  |  |
|  |  |  |  |  | Retired | 9.1% | x |  |  |
|  |  |  |  |  | Employed | 8.6% | x |  |  |
|  |  |  |  |  | Full-time employment | 7.2% | x |  |  |
| Weight and-or BMI recorded | Oct 2021 – Oct 2023 | 41.9% | 34 | 91.5% | Body mass index | 88.7% |  | x |  |
|  |  |  |  |  | Body weight | 10.3% |  | x |  |
|  |  |  |  |  | O/E - weight | 40.0% |  | x |  |
|  |  |  |  |  | BMI | 0.4% |  | x |  |
|  |  |  |  |  | Birth weight | 0.30% |  | x |  |
| Current smoker (recorded in last 24m) | Oct 2021 – Oct 2023 | 7.3% | 47 | 49.0% | Cigarette smoker | 45.8% |  | x |  |
|  |  |  |  |  | Current smoker | 18.7% |  | x |  |
|  |  |  |  |  | Smoker | 12.3% |  | x |  |
|  |  |  |  | 115.0% | Light cigarette smoker (1-9 cigs/day) | 7.9% |  | x |  |
|  |  |  |  |  | Moderate cigarette smoker (10-19 cigs/day) | 4.1% |  | x |  |
| Any smoking status (recorded in last 24m) | Oct 2021 – Oct 2023 | 44.8% | 117 | 92.3% | Never smoked tobacco | 53.7% |  | x |  |
|  |  |  |  |  | Ex-smoker | 15.6% |  | x |  |
|  |  |  |  |  | Cigarette smoker | 7.4% |  | x |  |
|  |  |  |  |  | Non-smoker | 5.9% |  | x |  |
|  |  |  |  |  | Current smoker | 3.0% |  | x |  |
| Alcohol consumption record | Oct 2021 – Oct 2023 | 18.0% | 39 | 61.6% | AUDIT-C (Alcohol Use Disorders Identification Test - Consumption) score | 43.4% |  | x |  |
|  |  |  |  |  | Alcohol units consumed per week | 35.1% |  | x |  |
|  |  |  |  |  | Alcohol use disorders identification test score | 35.1% |  | x |  |
|  |  |  |  |  | AUDIT-C score-freq drunk 6+units (fem)/8+units (male) last yr | 35.1% |  |  | x |
|  |  |  |  |  | Alcohol units consumed per day | 35.1% |  | x |  |
| Family history of neoplasm | Recorded ever | 14.8% | 479 | No comparator for coding prevalence of this data item sourced | FH: Cancer - * | 17.4% |  |  | x |
|  |  |  |  |  | FH: Neoplasm - * | 17.3% |  |  | x |
|  |  |  |  |  | FH: * - breast | 8.9% |  |  | x |
|  |  |  |  |  | Family history of cancer | 6.7% |  | x |  |
|  |  |  |  |  | FH: Breast cancer | 6.2% |  | x |  |
| Environmental pollutants | Oct 2021 – Oct 2023 | 0.04% | 45 | No comparator for coding prevalence of this data item sourced | Exposure to confirmed case of SARS-CoV-2 (severe acute respiratory syndrome coronavirus 2) infection | 17.8% |  | x |  |
|  |  |  |  |  | Exposure to Chlamydia trachomatis | 10.9% |  | x |  |
|  |  |  |  |  | Exposure to tuberculosis | 9.0% |  | x |  |
|  |  |  |  |  | Exposure to Mycobacterium tuberculosis | 6.1% |  | x |  |
|  |  |  |  |  | Close exposure to SARS-CoV-2 (severe acute respiratory syndrome coronavirus 2) infection | 6.1% |  | x |  |
| Fast Track Referral Coding | New episode added last 12 mnths | 4.1% | 34 | 78% | 2 week rule referral - breast | 81.1% |  |  | x |
|  |  |  |  |  | 2 week rule referral - skin | 68,1% |  |  | x |
|  |  |  |  |  | 2 week rule referral - colorectal | 65.0% |  |  | x |

*Table 10*. Theme 2**:** Completeness, code diversity and validation, and granularity of coding.

| **REPORT NAMES** | **Time Period** | **Completeness**  **and**  **Code diversity** | | **Code validation** | **Granularity** ​(Note: granularity - top 5 codes - if 5 were not available then min number used were reviewed) | | | | |
| --- | --- | --- | --- | --- | --- | --- | --- | --- | --- |
|  |  | Completeness - % of eligible patients with code captured | No of unique SNOMED/ EMIS codes in search | EMIS code capture coverage as a proportion of comparator | Code prevalence - TOP 5 | % or Total no. of all codes captured in NCL | SNOMED Parent | SNOMED child | Non-SNOMED |
| Cancer-Bowel - 60-74yrs did not return screening kit | Run date (end 2023?) - 2 years and 6 months | 34.6% | 4 | No comparator for coding prevalence for of kit DNR's, only uptake and coverage | No response to BCSP invitation | 89.1% |  |  | x |
|  |  |  |  |  | No response to bowel cancer screening programme invitation | 9.4% |  | x |  |
|  |  |  |  |  | Did not attend bowel cancer screening | 1.4% |  | x |  |
|  |  |  |  |  | Unlinked Report: No response to BCSP invitation | 0.0% |  | x |  |
| Cancer-Bowel - 60-74yrs screened | Run date (end 2023?) - 2 years and 6 months | 61.3% | 10 | 98.6% | BCSP FOB test normal | 90.4% |  |  | x |
|  |  |  |  |  | Bowel cancer screening programme faecal occult blood test normal | 4.7% |  | x |  |
|  |  |  |  |  | Bowel cancer screening - negative FOBs | 2.4% |  |  | x |
|  |  |  |  |  | BCSP FOB test abnormal | 1.6% |  |  | x |
| Cancer-Bowel abnormal result | Run date (end 2023?) - 2 years and 6 months | 1.3% | 5 | No comparator for coding prevalence for abnormal screening results, only uptake and coverage | BCSP FOB test abnormal | 84.7% |  |  | x |
|  |  |  |  |  | Bowel cancer screening programme faecal occult blood test abnormal | 7.7% |  | x |  |
|  |  |  |  |  | Bowel cancer screening - positive FOBs | 6.1% |  |  | x |
|  |  |  |  |  | Bowel cancer screening programme liquid faecal immunochemical test abnormal | 0.9% |  |  | x |
| Cancer-Breast - 50-70yrs screened | Run date (end 2023?) - 3 years and 6 months | 45.1% | 30 | 78.8% | Mammography normal | 39.2% | x |  |  |
|  |  |  |  |  | Breast neoplasm screening normal | 38.2% | x |  |  |
|  |  |  |  |  | Attended breast screening clinic | 10.9% | x |  |  |
|  |  |  |  |  | Mammography | 3.7% | x |  |  |
| Cancer-Breast - 50-70yrs abnormal result | Run date (end 2023?) - 3 years and 6 months | 1.4% | 7 | No comparator for coding prevalence for abnormal screening results, only uptake and coverage | Mammography abnormal | 64.6% |  | x |  |
|  |  |  |  |  | Breast neoplasm screening abnormal | 30.6% |  | x |  |
|  |  |  |  |  | Breast neoplasm screen abnorm | 1.9% |  | x |  |
|  |  |  |  |  | Breast screen abnorm.-told pat | 1.5% |  |  | x |
| Cancer-Breast - 50-70yrs normal result | Run date (end 2023?) - 3 years and 6 months | 43% | 3 | No comparator for coding prevalence for normal screening results, only uptake and coverage | Mammography normal | 52% |  | x |  |
|  |  |  |  |  | Breast neoplasm screening normal | 42.3% |  | x |  |
|  |  |  |  |  | Breast neoplasm screen normal | 5.7% |  | x |  |
| Cancer-Breast cancer detected | Run date (end 2023?) - 3 years and 6 months | 0.5% | 35 | 124.9% | Malignant neoplasm of female breast | 62.2% |  | x |  |
|  |  |  |  |  | Malignant tumour of breast | 13.9% |  | x |  |
|  |  |  |  |  | Carcinoma of breast | 6.5% |  | x |  |
|  |  |  |  |  | Carcinoma in situ of breast | 3.9% |  | x |  |
| Cancer-Cervical - 25-49y adequate smear | Run date (end 2023?) - 3 years and 6 months | 57.6% | 103 | 99.8% | Cervical smear - human papillomavirus negative | 27.8% |  | x |  |
|  |  |  |  |  | HPV - Human papillomavirus test negative | 22.8% |  | x |  |
|  |  |  |  |  | Liquid based cervical cytology screening | 14% |  | x |  |
|  |  |  |  |  | Cervical smear - negative | 12.9% |  | x |  |
| Cancer-Cervical - 50-64y adequate smear | Run date (end 2023?) - 5 years and 6 months | 69.8% | 98 | 98.3% | HPV - Human papillomavirus test negative | 24.2% |  | x |  |
|  |  |  |  |  | Cervical smear - human papillomavirus negative | 22.1% |  | x |  |
|  |  |  |  |  | Liquid based cervical cytology screening | 12.7% |  | x |  |
|  |  |  |  |  | Cervical smear - negative | 10.0% |  | x |  |

*Table 11* *.* Theme 3**:** Completeness, code diversity and validation, and granularity of coding.

| **REPORT NAME** | **Time Period** | **Completeness**  **and**  **Code diversity** | | **Code validation** | **Granularity** | | | | |
| --- | --- | --- | --- | --- | --- | --- | --- | --- | --- |
|  |  | Completeness - % of eligible patients with code captured | No of unique SNOMED/ EMIS codes in search | EMIS code capture coverage as a proportion of comparator | Prevalence for top 5 codes | % or Total no. of all codes captured in NCL | SNOMED Parent | SNOMED child | Non-SNOMED |
| Malignant Neoplastic Disease | New episode added last 12 months | 0.3% | 677 | 73% | Basal cell carcinoma of skin | 13.7% |  | x |  |
|  |  |  |  |  | Malignant tumour of prostate | 11.2% |  | x |  |
|  |  |  |  |  | Malignant neoplasm of female breast | 7.0% |  | x |  |
|  |  |  |  |  | Adenocarcinoma of prostate | 4.5% |  | x |  |
|  |  |  |  |  | Squamous cell carcinoma of skin | 3.8% |  | x |  |
| Malignancy Stage | New episode added last 12 months | 0.01% | 4 | 0.2% | Infiltrating ductal carcinoma of breast, stage 1 | 33.3% |  | x |  |
|  |  |  |  |  | Infiltrating ductal carcinoma of breast, stage 2 | 33.3% |  | x |  |
|  |  |  |  |  | Malignant melanoma stage IA | 16.7% |  | x |  |
|  |  |  |  |  | Lymphoma stage IV | 16.7% |  | x |  |
| Treatment regime | New episode added last 12 months | 0.03% | 35 | 3% | Chemotherapy | 25.7% |  | x | x |
|  |  |  |  |  | Radiotherapy completed | 18.7% |  | x |  |
|  |  |  |  |  | Chemotherapy cycle | 8.9% |  | x |  |
|  |  |  |  |  | Radiation oncology AND/OR radiotherapy | 5.4% |  |  |  |
|  |  |  |  |  | Excision of basal cell carcinoma | 5.4% |  |  |  |
| Cancer Care Review | New episode added last 12 months | 0.3% | 1 |  | one code used |  |  |  |  |
| Has a carer | Recorded ever | 1.2% | 17 | No comparator for coding prevalence of this data item sourced | Has a carer | 80.1% |  | x |  |
|  |  |  |  |  | Has an informal carer | 6.8% |  | x |  |
|  |  |  |  |  | Has a paid carer | 6.3% |  | x |  |
|  |  |  |  |  | Has a parent carer | 3.1% |  | x |  |
|  |  |  |  |  | Has voluntary carer | 1.1% |  | x |  |

*Table 12. Personalised cancer care metrics - Enfield and NCL - as described in the methods, additional searches run at Enfield level and compared with the HealtheIntent NCL level data, that only covered completion of codes*

| Report name | Enfield data | | HEI data NCL |
| --- | --- | --- | --- |
| Personalised care interventions | Patients | % prevalence against adult population | % prevalence in NCL Cancer Registry |
| Cancer care plan given | 11 | 1% | 1% |
| Cancer End of Treatment Summary Plan | <5 | 1% | 1% |
| Holistic Needs Assessment | 31 | 2% | 2% |

*ITEM 1*: Revised Manuscript - Tracked version:

*ITEM 2:* Revised Supplementary – Tracked version:
